# Supplementary material for: Divergent WNT signaling and drug sensitivity profiles within hepatoblastoma tumors and organoids
Source: Nat Commun. 2024 Nov 20;15:8576. doi: 10.1038/s41467-024-52757-w (PMC11579375; doi:10.1038/s41467-024-52757-w)
Supplement: Supplementary file 1 — Supplementary Information [file 41467_2024_52757_MOESM1_ESM.pdf]

Supplementary Information

Divergent WNT Signaling and Drug Sensitivity Profiles within Hepatoblastoma Tumors and Organoids

Kluiver, Lu *et al.*

Supplementary Figures

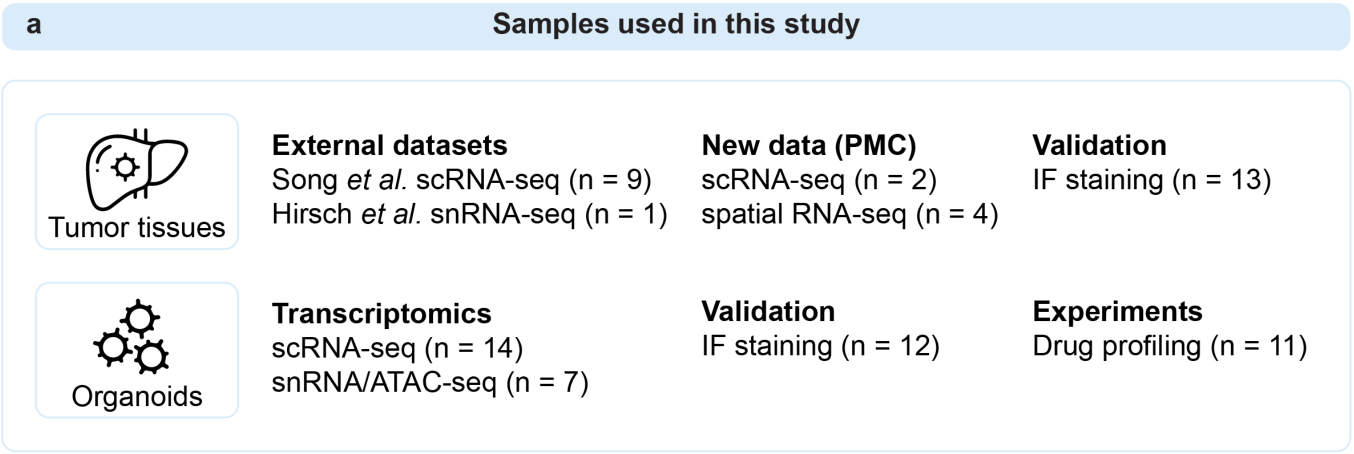

Supplementary Figure 1. Single-cell hepatoblastoma processing and analysis.
(a) Overview of number of samples per analyses used in the study.

b **Reanalysis of Song *et al.***

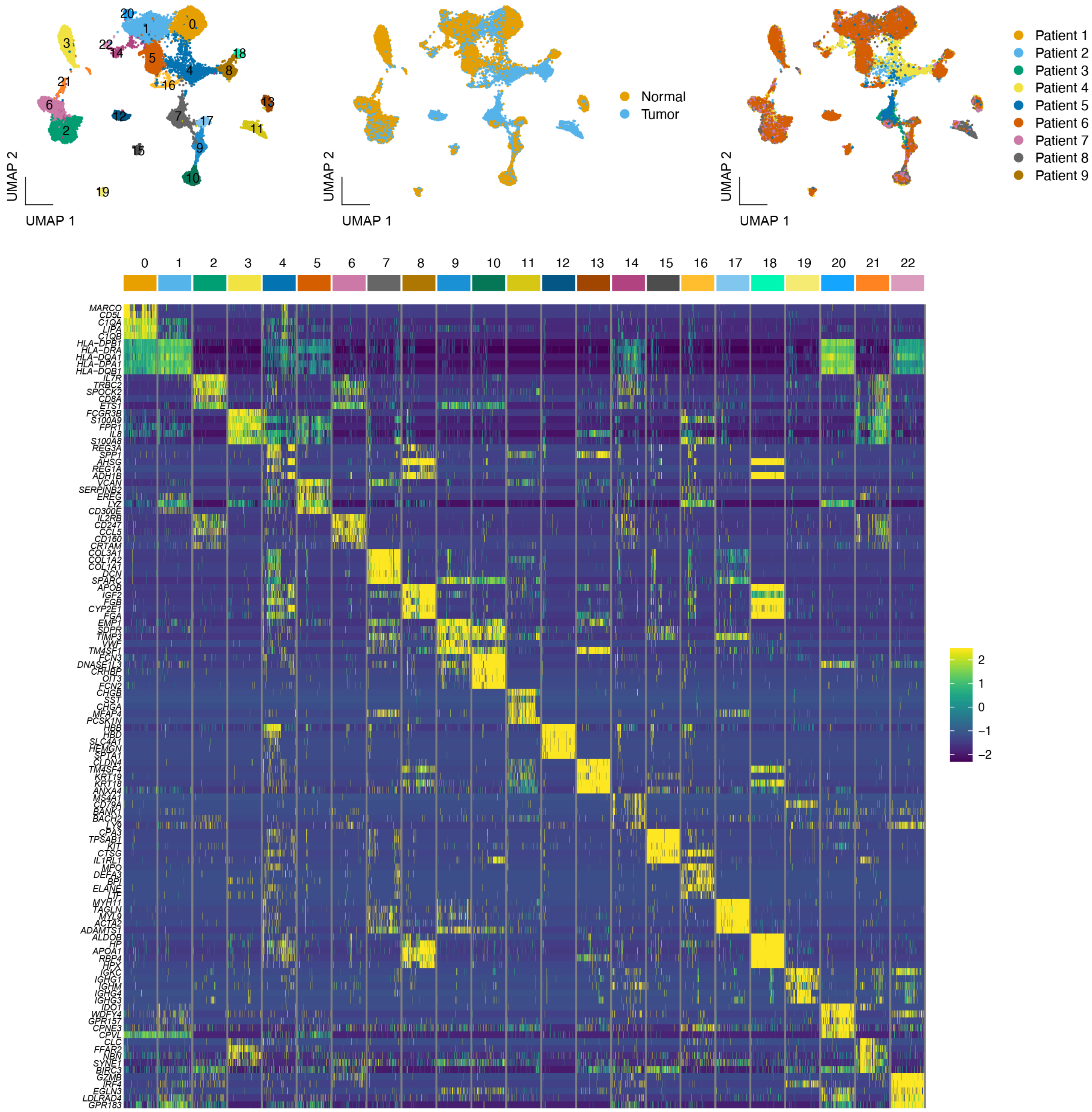

Figure legend is provided on the next page.

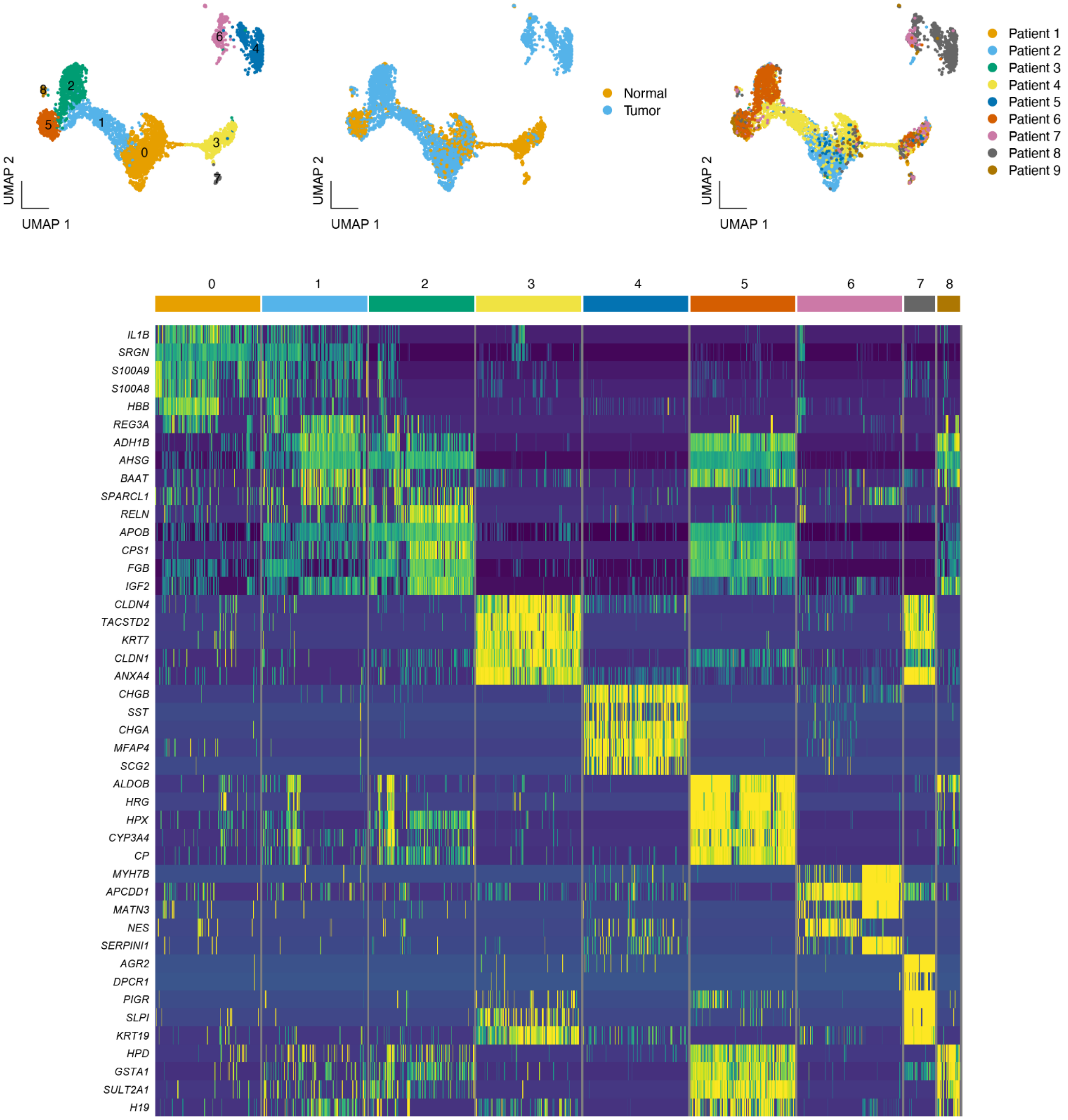

(b) UMAP showing the full dataset from Song *et al.* after data filtering, normalization, scaling, DecontX, fastMNN batch correction, dimensional reduction, and clustering, with a heatmap showing the top 5 differentially expressed markers per cluster (*previous page*). The epithelial normal and tumor clusters 4, 8, 11, 13 and 18 were subsetted and unbiased clustering was performed. UMAP showing the reprocessed subsetted clusters, with a heatmap showing the top 5 differentially expressed markers per cluster of the subsetted object (*this page*). We removed low-quality clusters likely contaminated with non-parenchymal cells, and the neuroendocrine cluster (4) for the final object. Clusters 2, 3, 5, 6, 7 and 8 were retained.

c **Heatmap of differentially expressed genes**

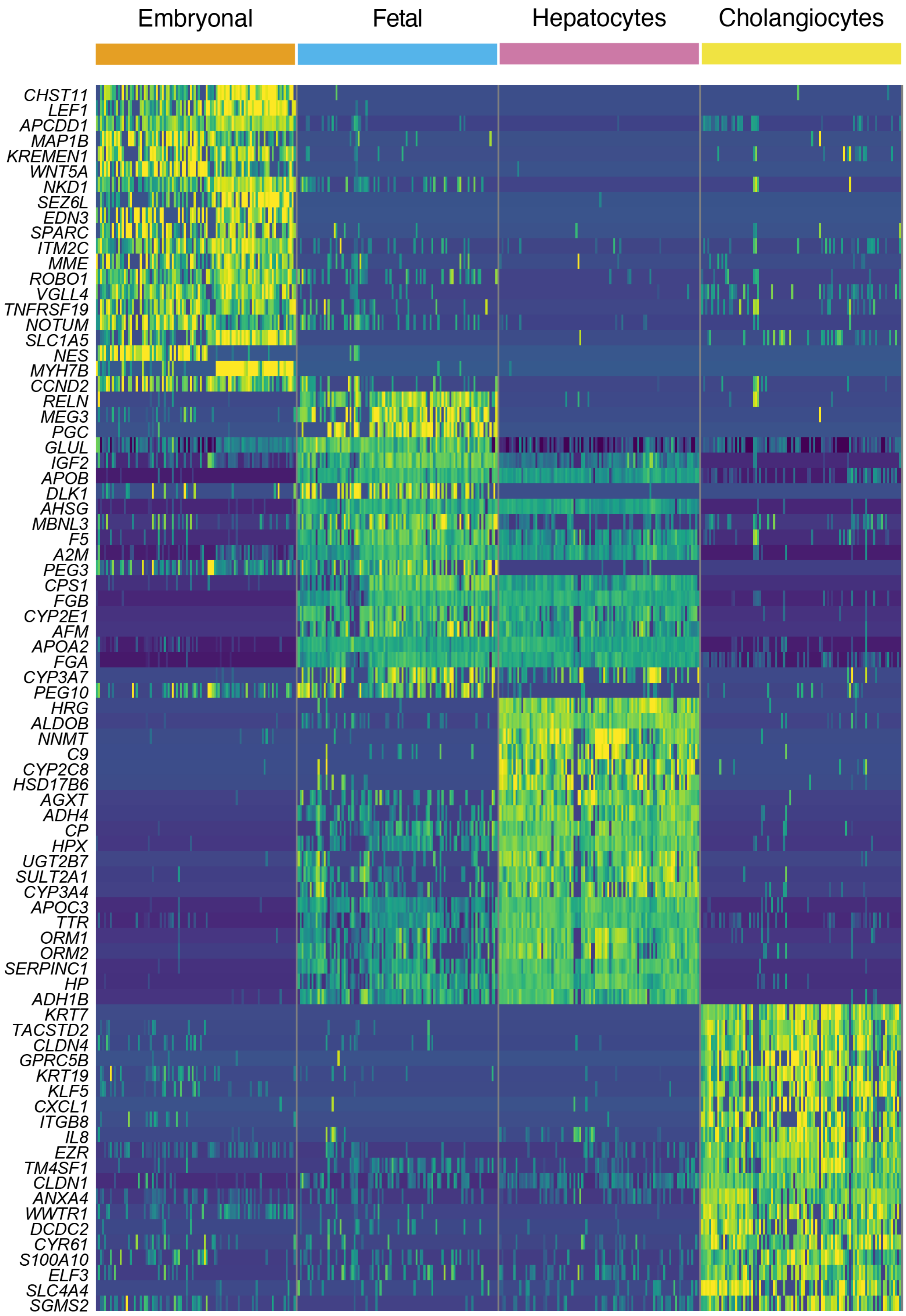

(c) Heatmap showing top differentially expressed genes of the final clusters.

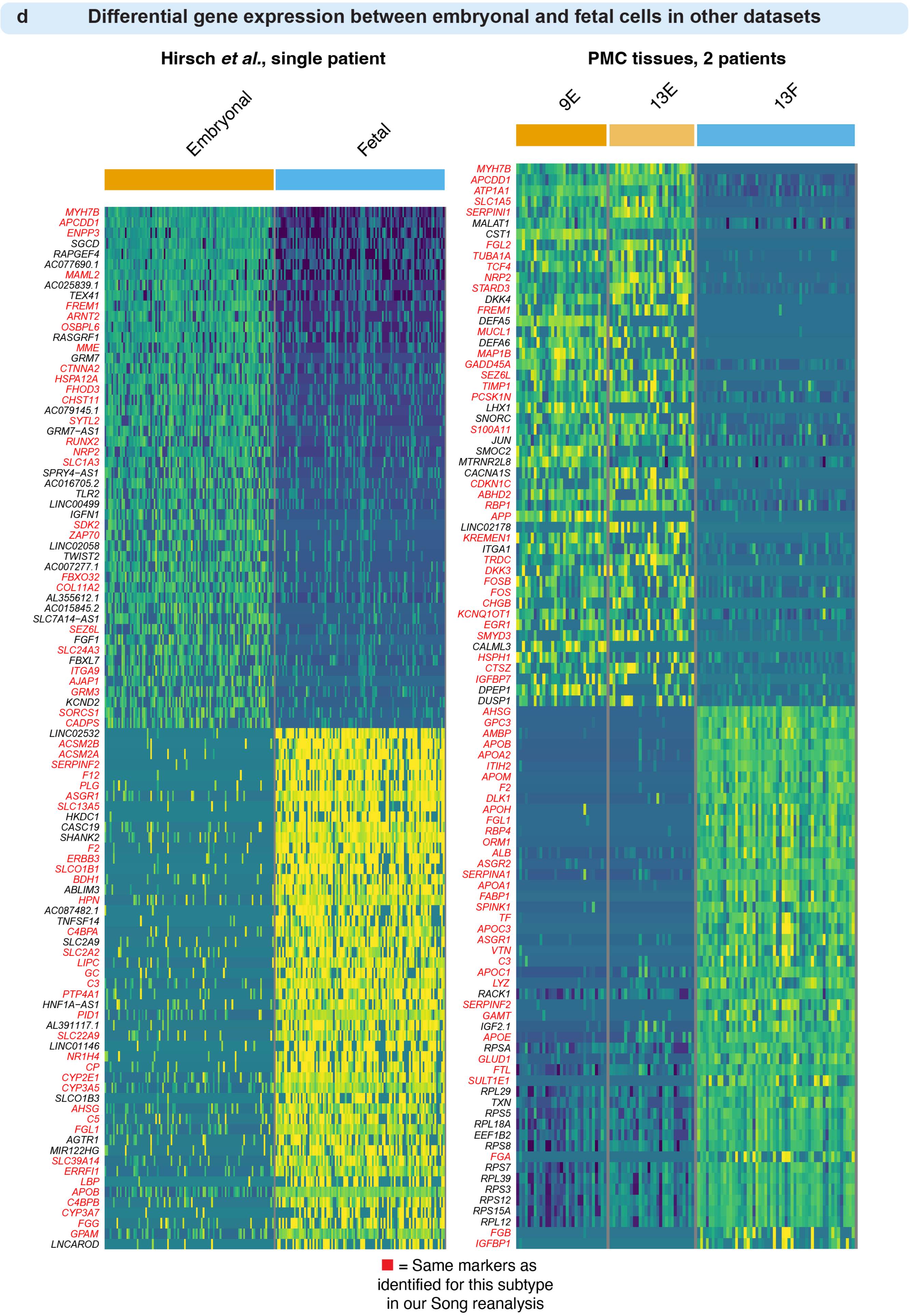

(d) Heatmaps showing the top 50 differentially expressed gene for both the Hirsch *et al.* and PMC samples. Genes which overlap with the respective cluster in the Song *et al.* reanalysis are marked in red.

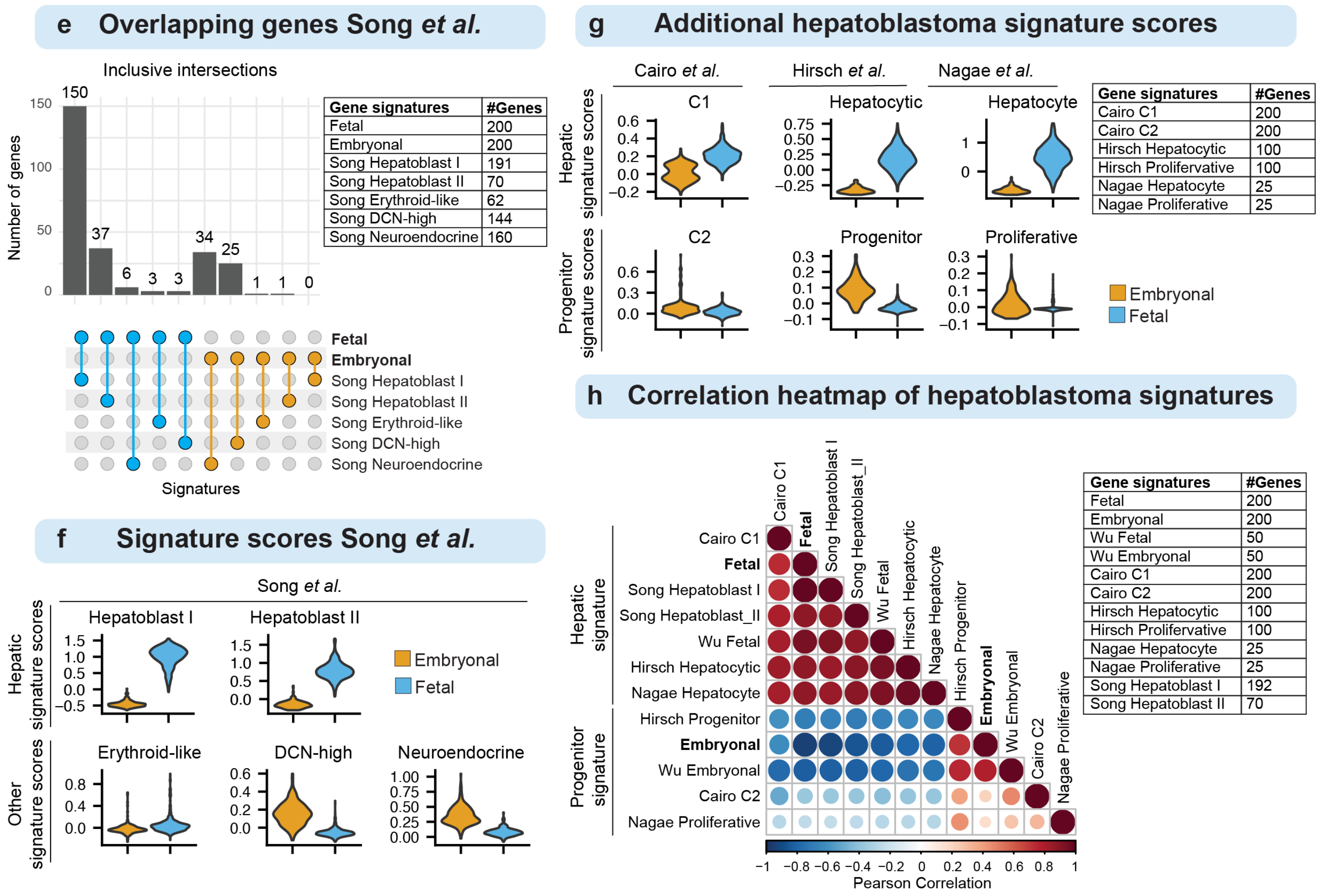

(e) Inclusive intersections between our gene signatures (top 200 DEGs) and the five single-cell RNA-sequencing tumor signatures described by Song *et al.*, visualized in an UpSet plot.
(f) Violin plot of tumor cell subsets showing scores of the different hepatoblastoma gene signatures from Song *et al*. (63-191 DEGs).
(g) Violin plot of tumor cell subsets showing scores of the different hepatoblastoma classifications based on gene signatures described by Cairo *et al.* (top 200 DEGs) and Hirsch *et al.* (top 100 DEGs), and Nagae *et al.* (top 25 DEGs).
(h) Correlation heatmap of the gene signature expression in tumor cell subset, grouped via complete linkage hierarchical clustering. The Pearson correlations between gene signatures were calculated using log-normalized gene expression values and z-scored to allow cross-signature comparisons. Only significant (adjusted p-value < 0.05) correlations are shown.

i Overlapping genes additional hepatoblastoma signatures

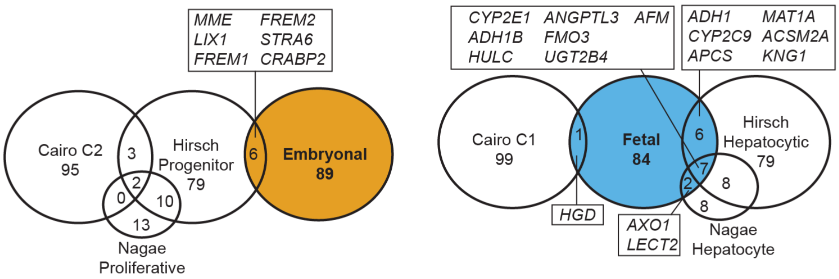

Gene signatures	#Genes
Fetal-like	100
Embryonal-like	100
Cairo C1	100
Cairo C2	100
Hirsch Hepatocytic	100
Hirsch Prolifervative	100
Nagae Hepatocyte	25
Nagae Proliferative	25

j Enrichment of general WNT genes

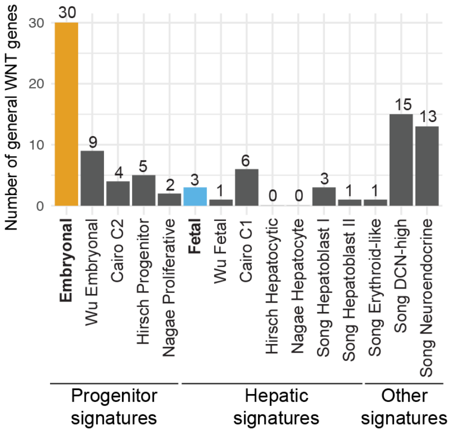

Gene signatures	#Genes
Embryonal	200
Wu Embryonal	50
Cairo C2	200
Hirsch Progenitor	100
Nagae Proliferative	25
Fetal	200
Wu Fetal	50
Cairo C1	200
Hirsch Hepatocytic	100
Nagae Hepatocyte	25
Song Hepatoblast I	191
Song Hepatoblast II	70
Song Erythroid-like	62
Song DCN-high	144
Song Neuroendocrine	160

General WNT gene list (n = 581)

(i) Graphical representation of overlapping genes between the different transcriptomic hepatoblastoma signatures.

(j) Enrichment of general WNT genes in the different transcriptomic hepatoblastoma signatures. Full list of genes is shown in **Supp. Data 3**.

k **Heatmap of differentially active SCENIC regulons**

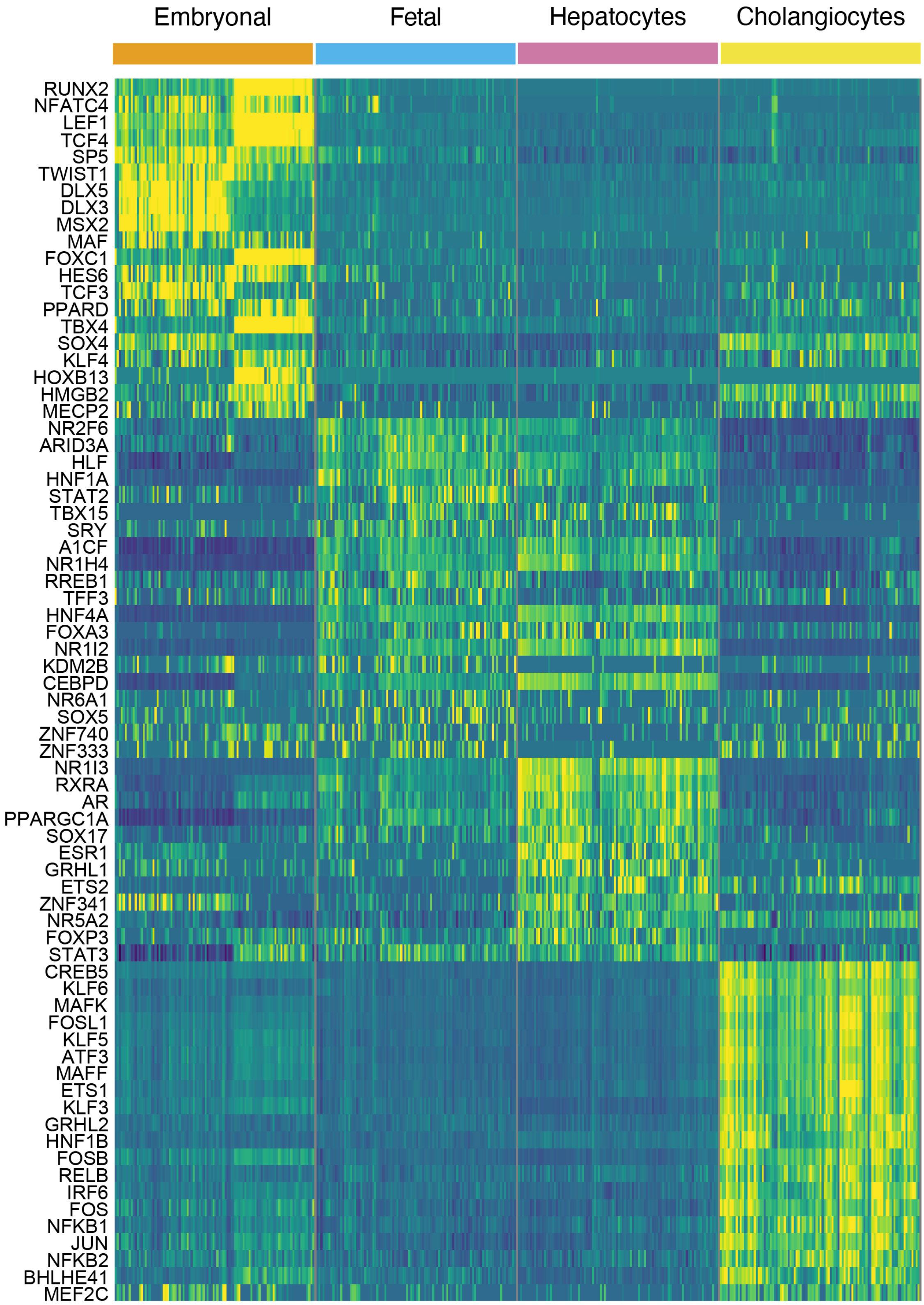

(k) Single-cell heatmap showing differentially active transcription factor regulons, using SCENIC, for each of the tumor clusters as well as normal hepatocytes and cholangiocytes.

l **Expression of selected markers in tissue, split by patient**

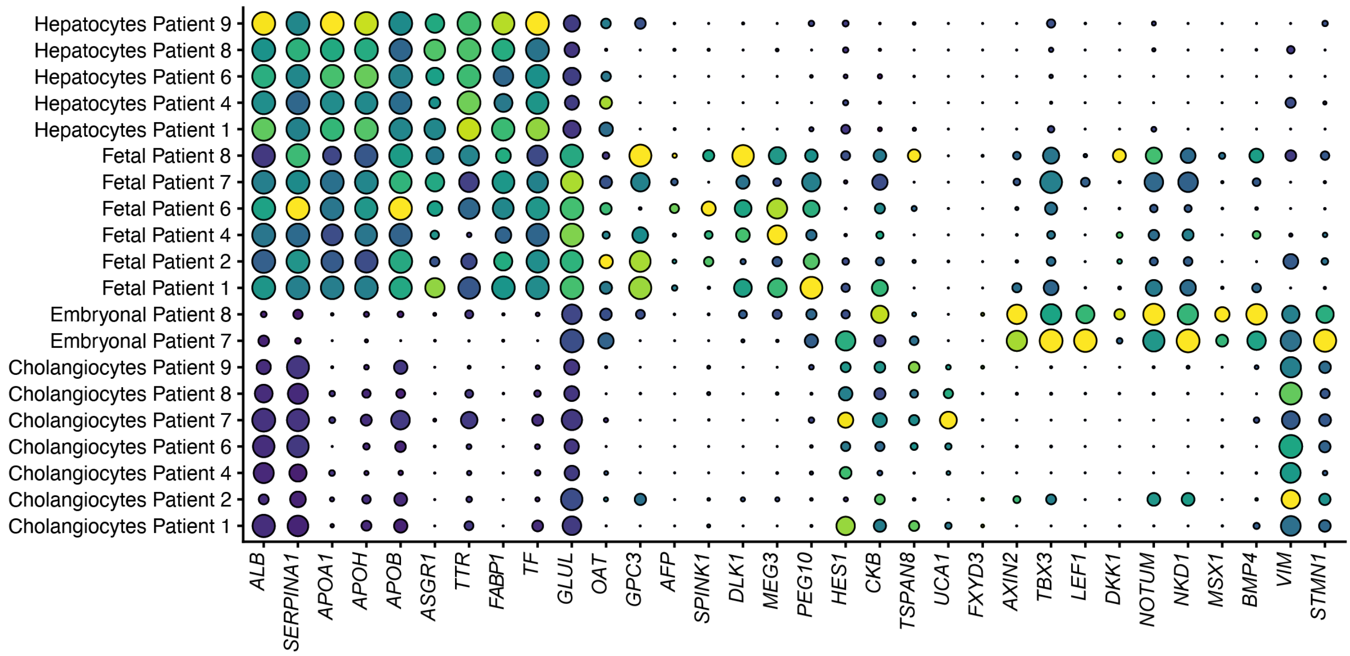

m **Regulon activity per cell type**

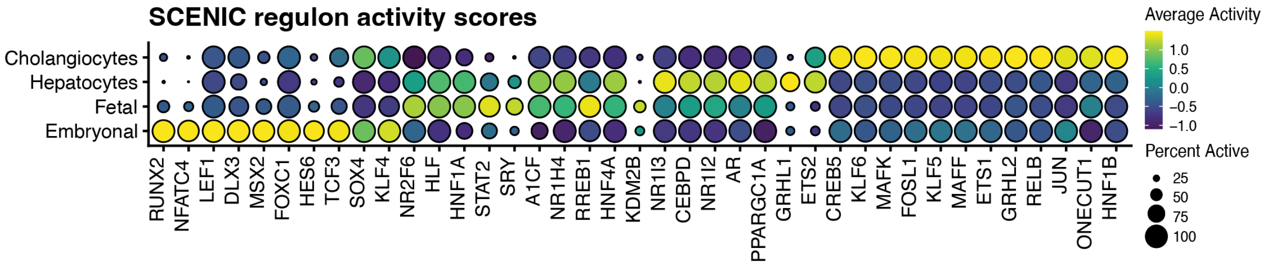

(l) Dot plot showing selected marker expression per cluster, per patient. Only populations with at least 10 cells are shown.
(m) Dot plot showing regulon activity per cell type.

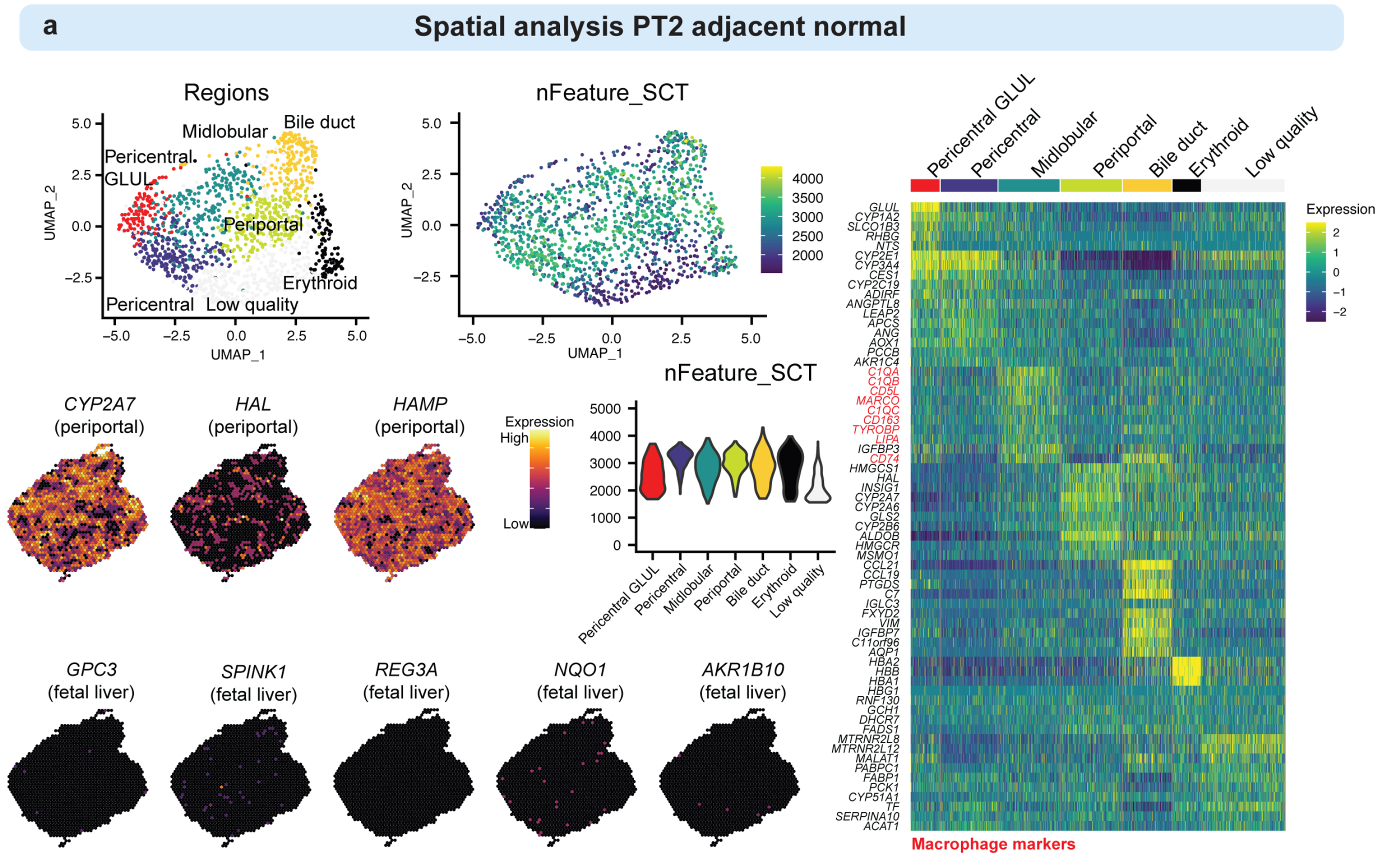

Supplementary Figure 2. Spatial transcriptomic analysis of adjacent normal liver and four hepatoblastoma tissues.

For each section individually, we inspected the H&E stainings and performed: (1) unsupervised graph-based clustering, (2) quality control assessment (SCTransformed features per cluster/spot visualized in UMAP representation and violin plot), (3) differential gene expression analysis (heatmap visualizing the top 10 differentially expressed genes per cluster) and (4) marker gene expression (visualized in violin plots or spatial distribution).

(a) Distal normal liver tissue from patient PT2 identified *GLUL* pericentral, pericentral, midlobular, periportal and bile duct regions. Quality control identified one cluster, assigned as “Low quality” (“nFeatures_SCT”<2000), which was excluded from further downstream analysis.

b **Spatial analysis PT2 hepatoblastoma**

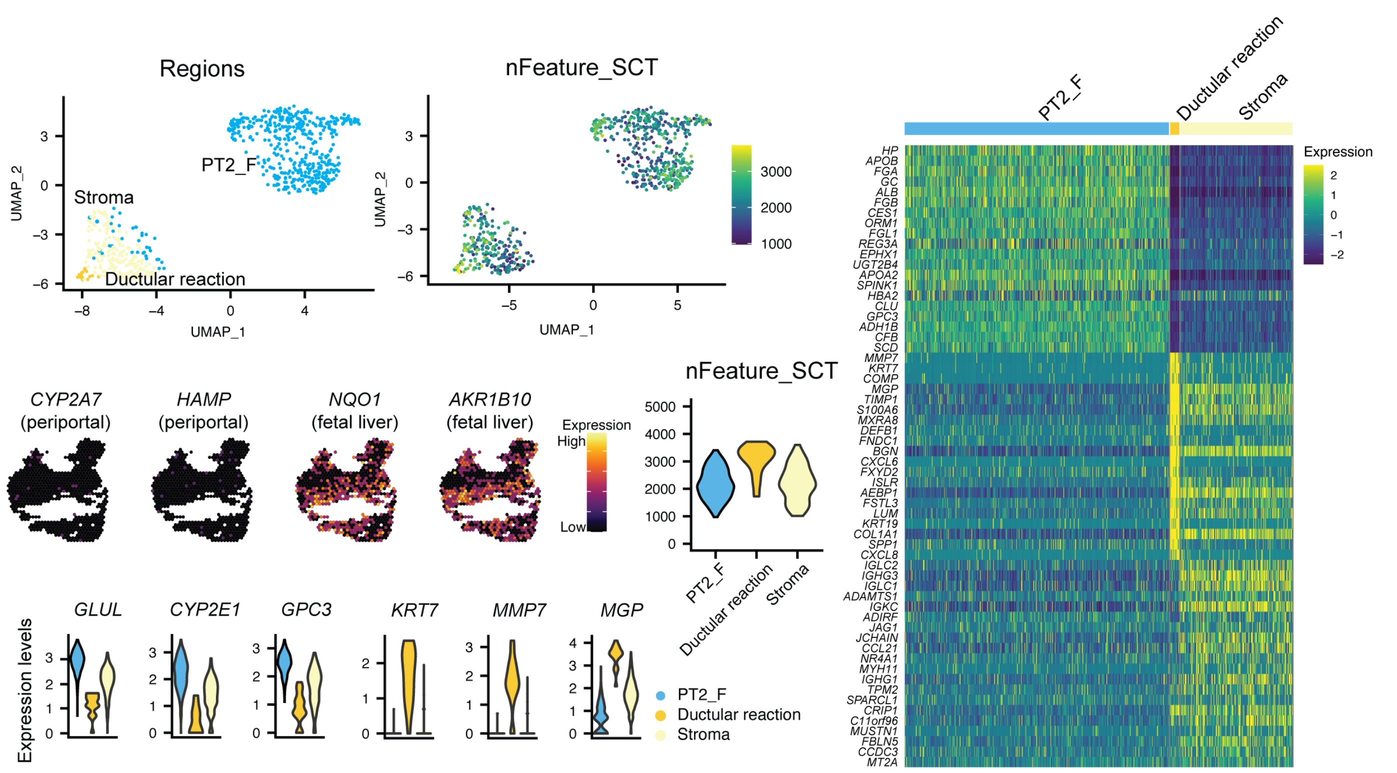

(b) The tumor of PT2 showed expressed of pericentral hepatocyte and fetal liver markers, but absence of periportal marker. Stromal regions expressed immune and endothelial markers (*CCL21*, *CCL19, MGP*). Expression of cholangiocyte markers indicates ductular reaction in the tumor stroma region.

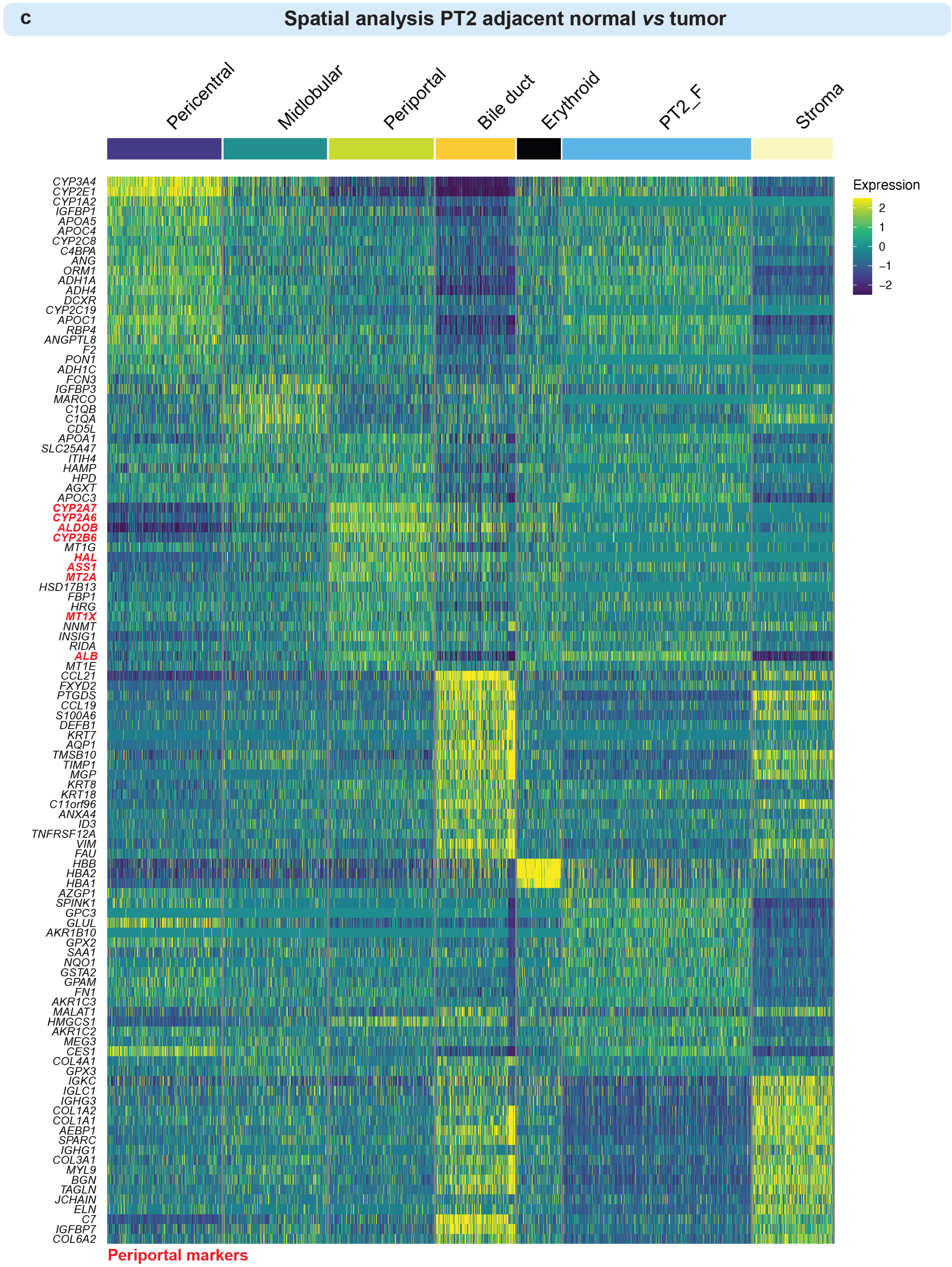

(c) Heatmap of differentially expressed genes between the clusters of the PT2 tumor and distal normal sections. Tumor spots expressed pericentral hepatocyte and fetal liver markers but showed reduced expression of periportal markers (marked in red).

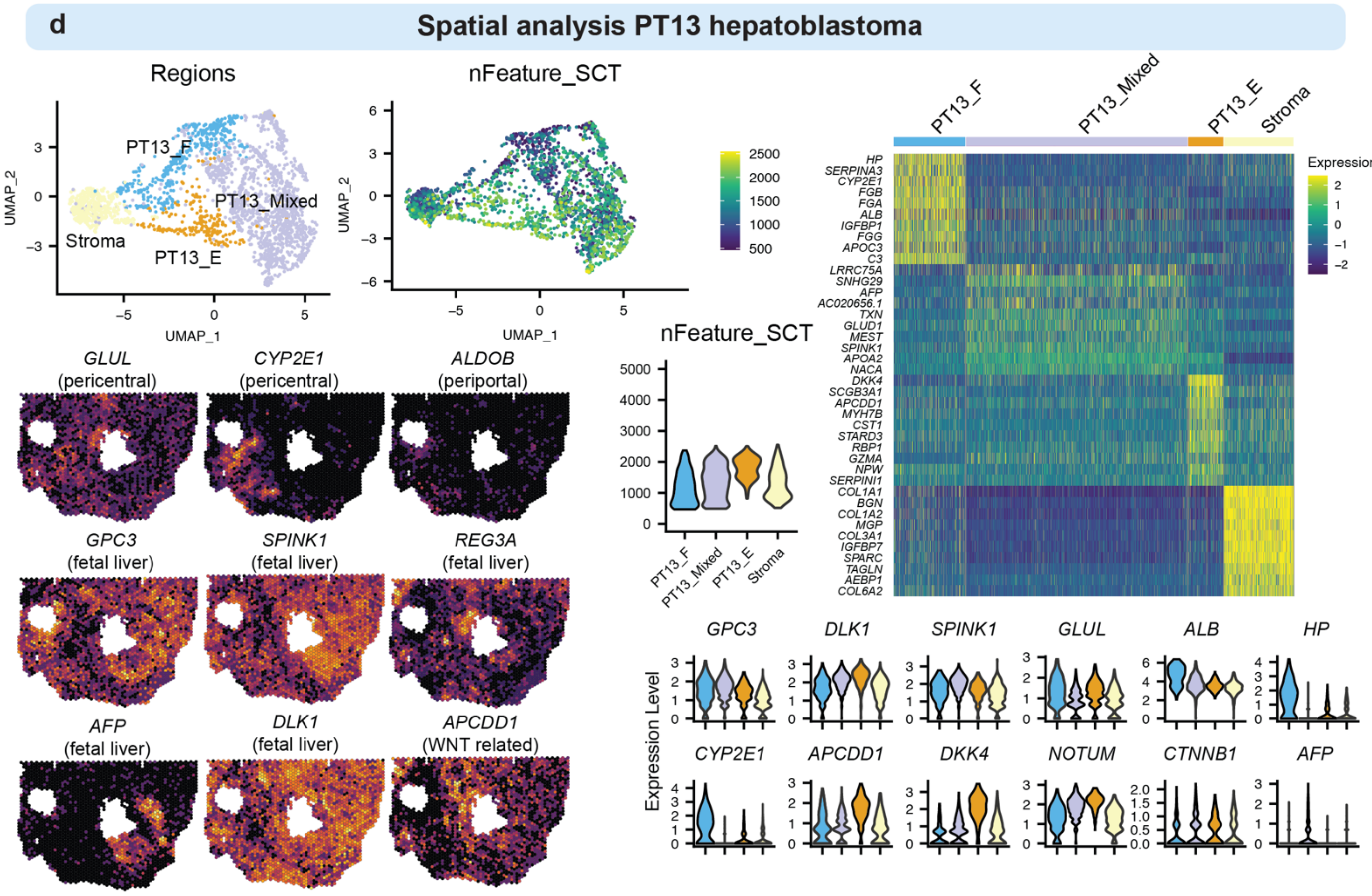

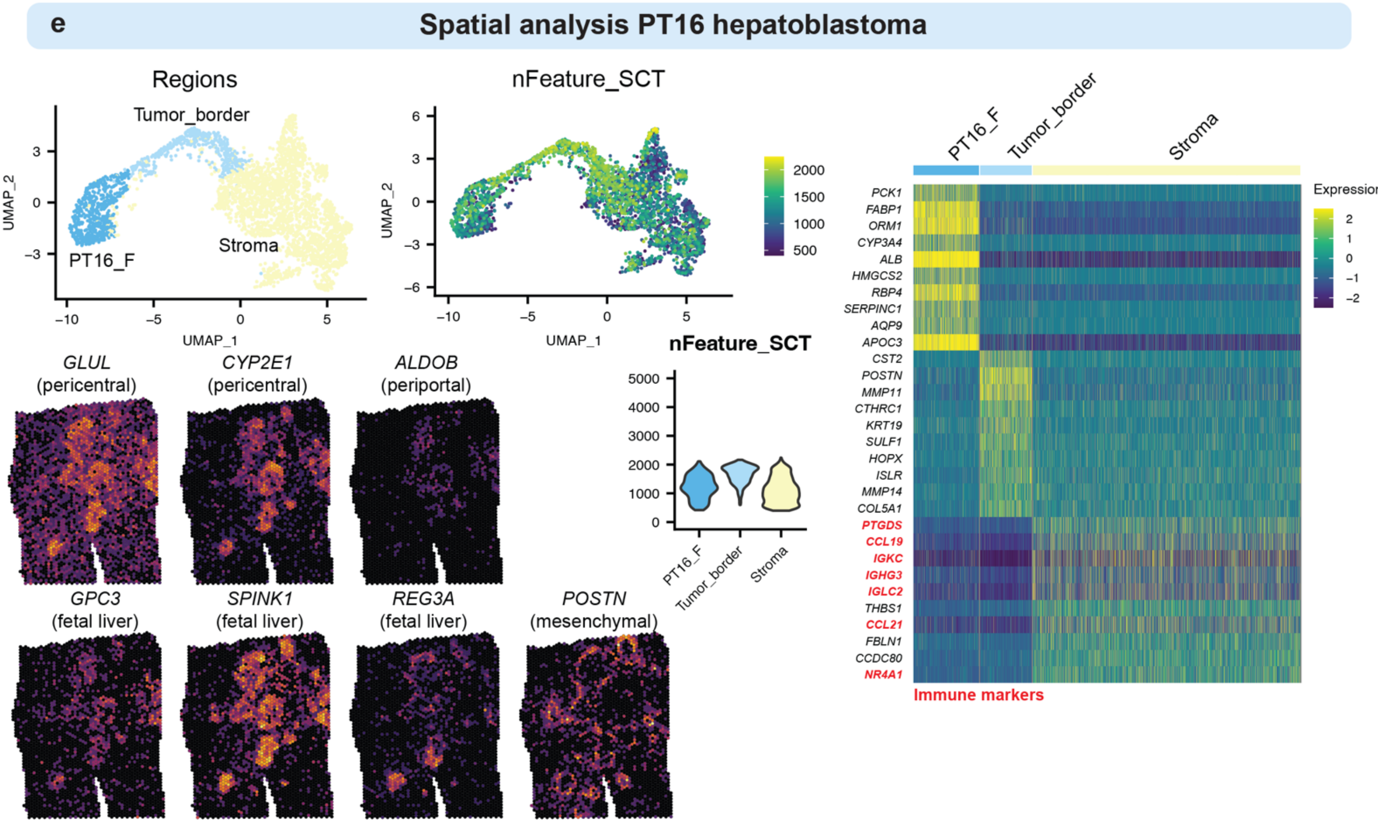

(d) The tumor of PT13 did not receive chemotherapy prior to resection. At least three different tumor clusters were identified: fetal-enriched, embryonal-enriched, and regions likely containing a mix of both populations of tumor cells. Additional tissue heterogeneity could be observed based on markers such as *AFP*.

(e) The tumor of PT16 contained fetal tumor regions. The tumor border showed a distinct expression profile, with high levels of *POSTN* (mesenchymal marker) and *KRT19* (cholangiocyte marker).

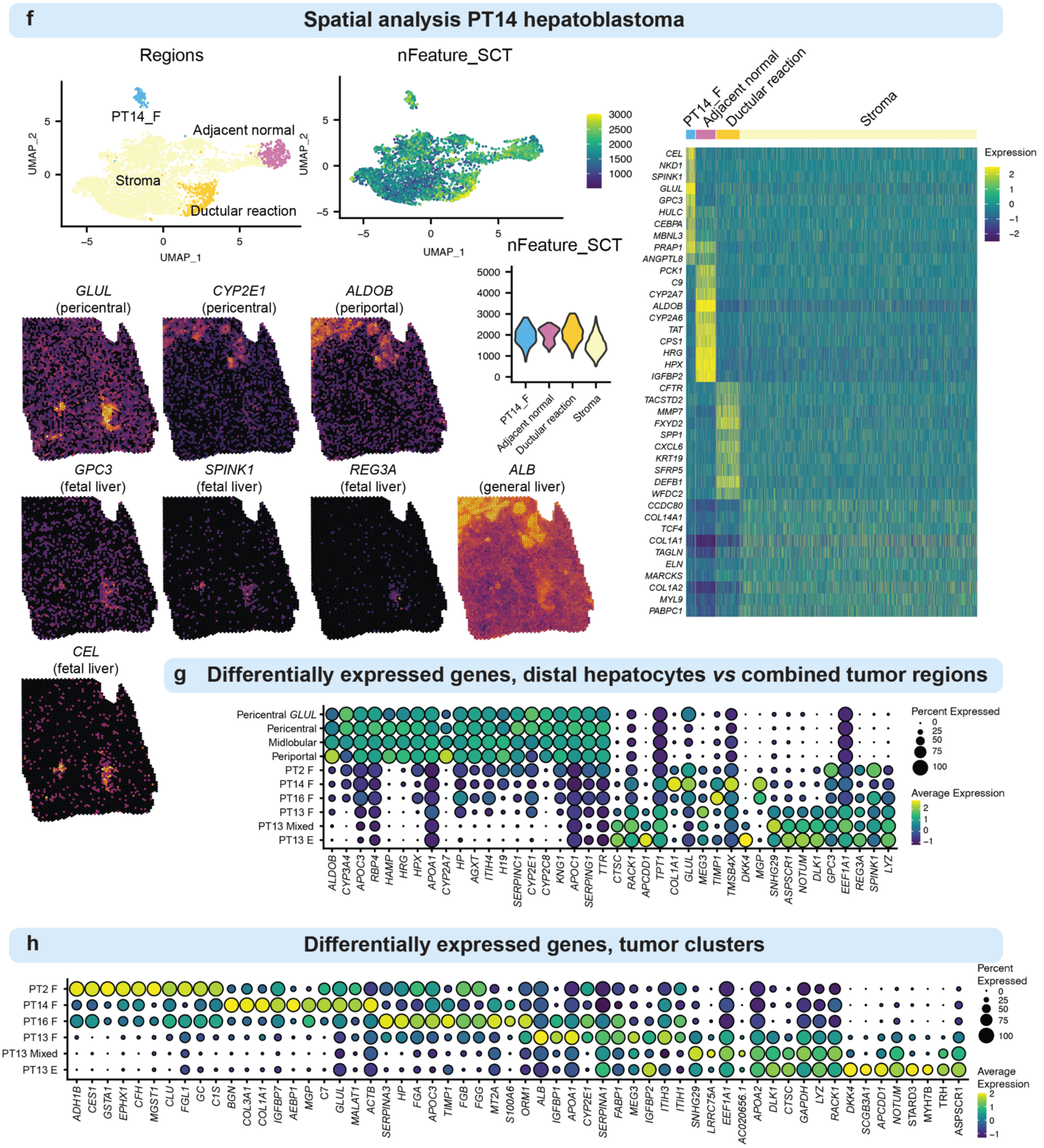

(f) The tumor of PT14 contained fetal tumor, normal liver, ductular reaction and stroma.

(g) Dot plot of the top 20 differentially expressed genes between distal hepatocytes (hepatocyte clusters from Normal PT2) and the combined tumor regions. There is an increased expression of fetal liver genes and a decreased expression of periportal markers in the tumor regions. Inter-tumor heterogeneity can also be observed.

(h) Dot plot of the top 10 differentially expressed genes between the tumor clusters, illustrating tumor-specific expression profiles, and additional heterogeneity within the PT13 tumor.

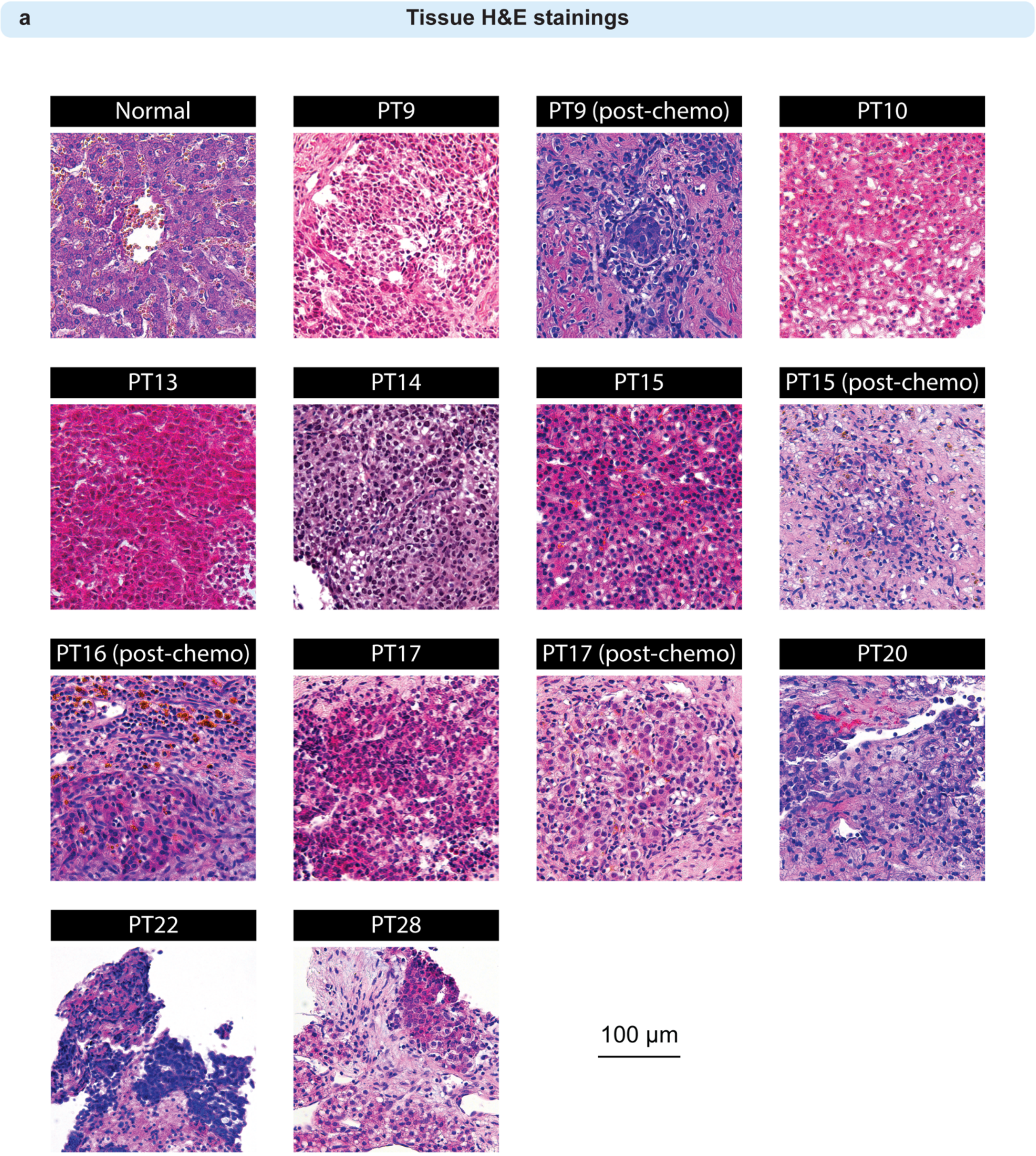

Supplementary Figure 3. Histological and immunofluorescent analysis of hepatoblastoma tissues.
(a) H&E stainings of tumor tissues.

b Tissue IF staining of LEF1, HNF4A and β-catenin

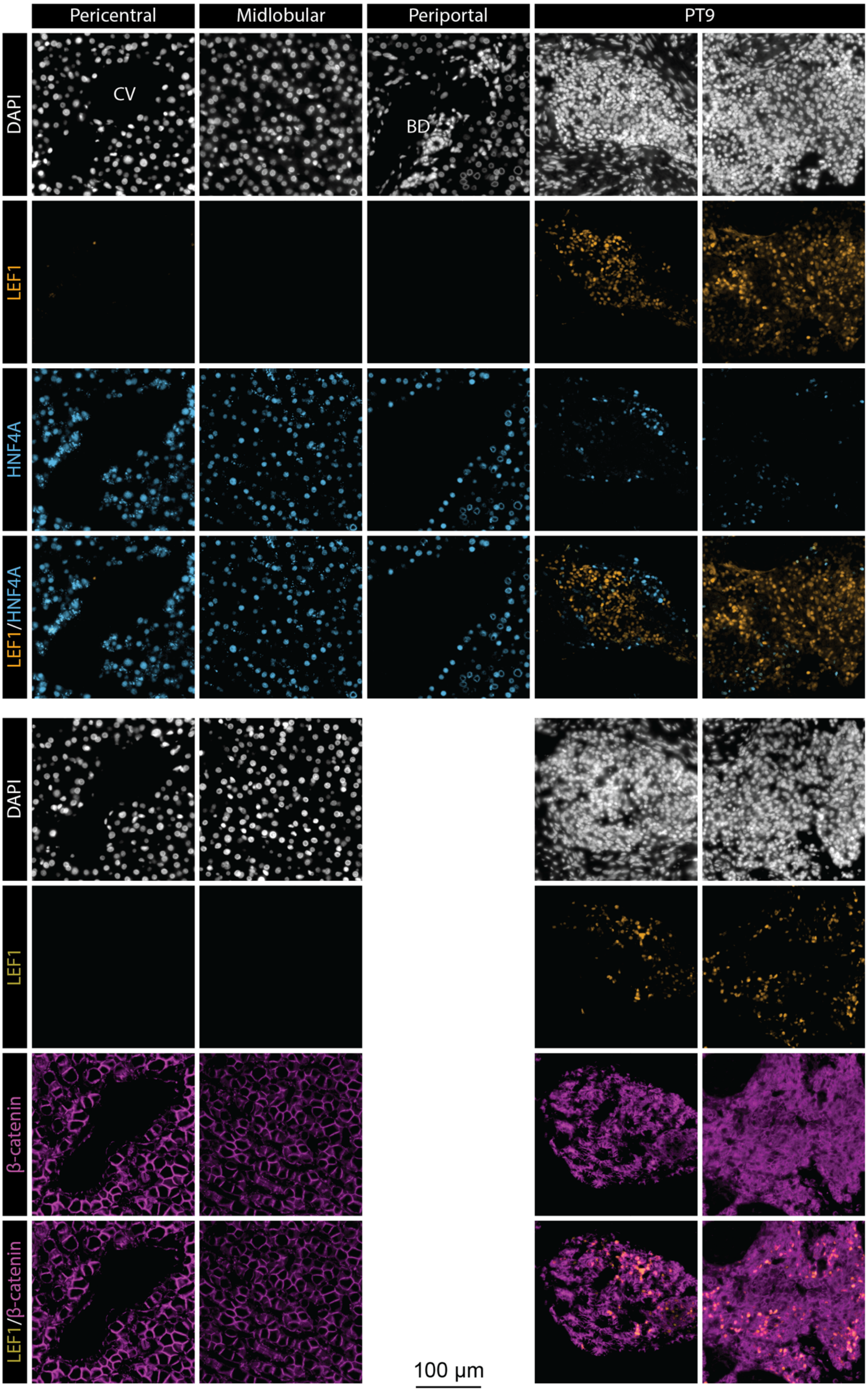

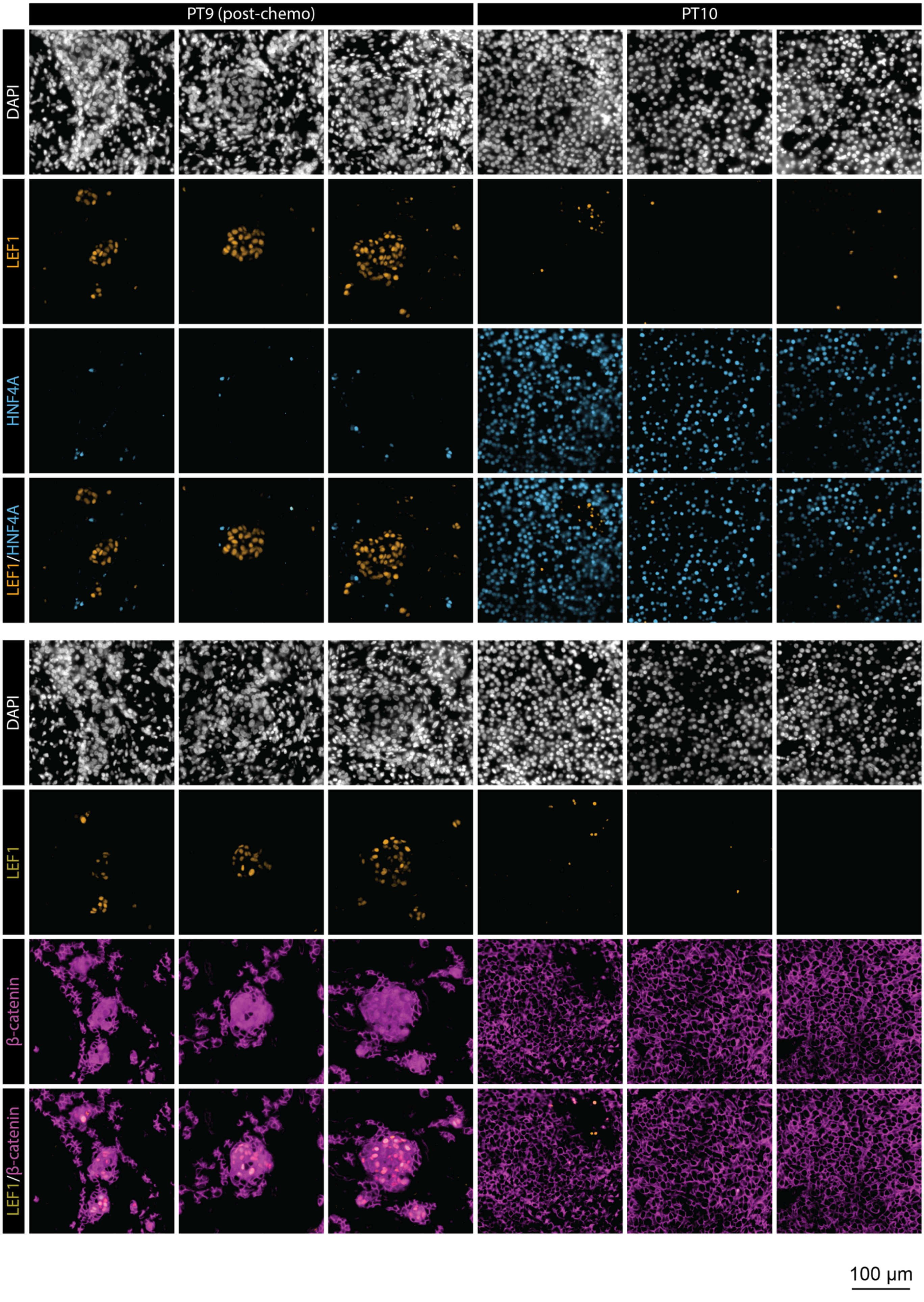

PT9 (post-chemo)
PT10
DAPI
LEF1
HNF4A
LEF1/HNF4A
DAPI
LEF1
β-catenin
LEF1/β-catenin
100 μm

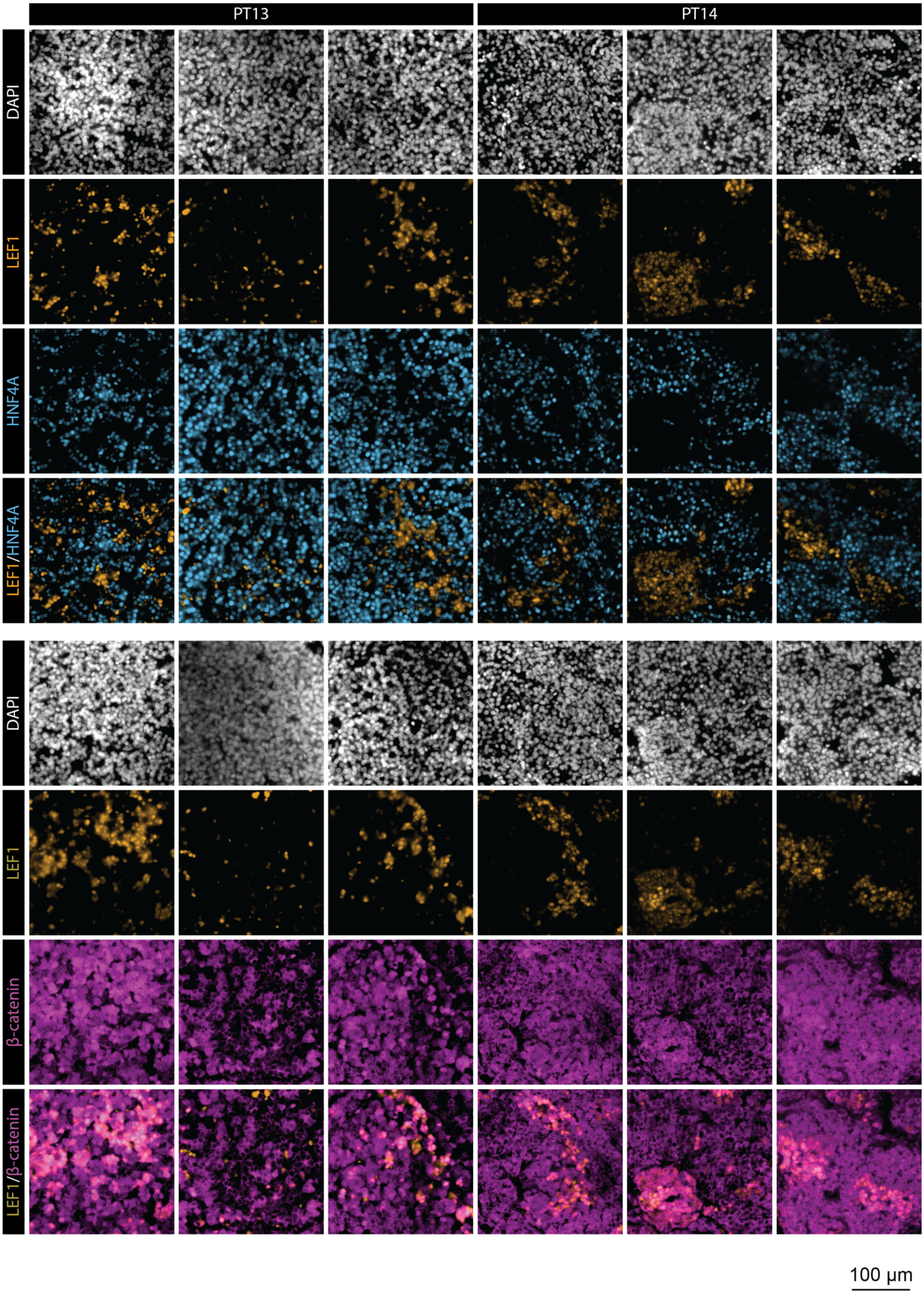

PT13
PT14
DAPI
LEF1
HNF4A
LEF1/HNF4A
DAPI
LEF1
β-catenin
LEF1/β-catenin
100 µm

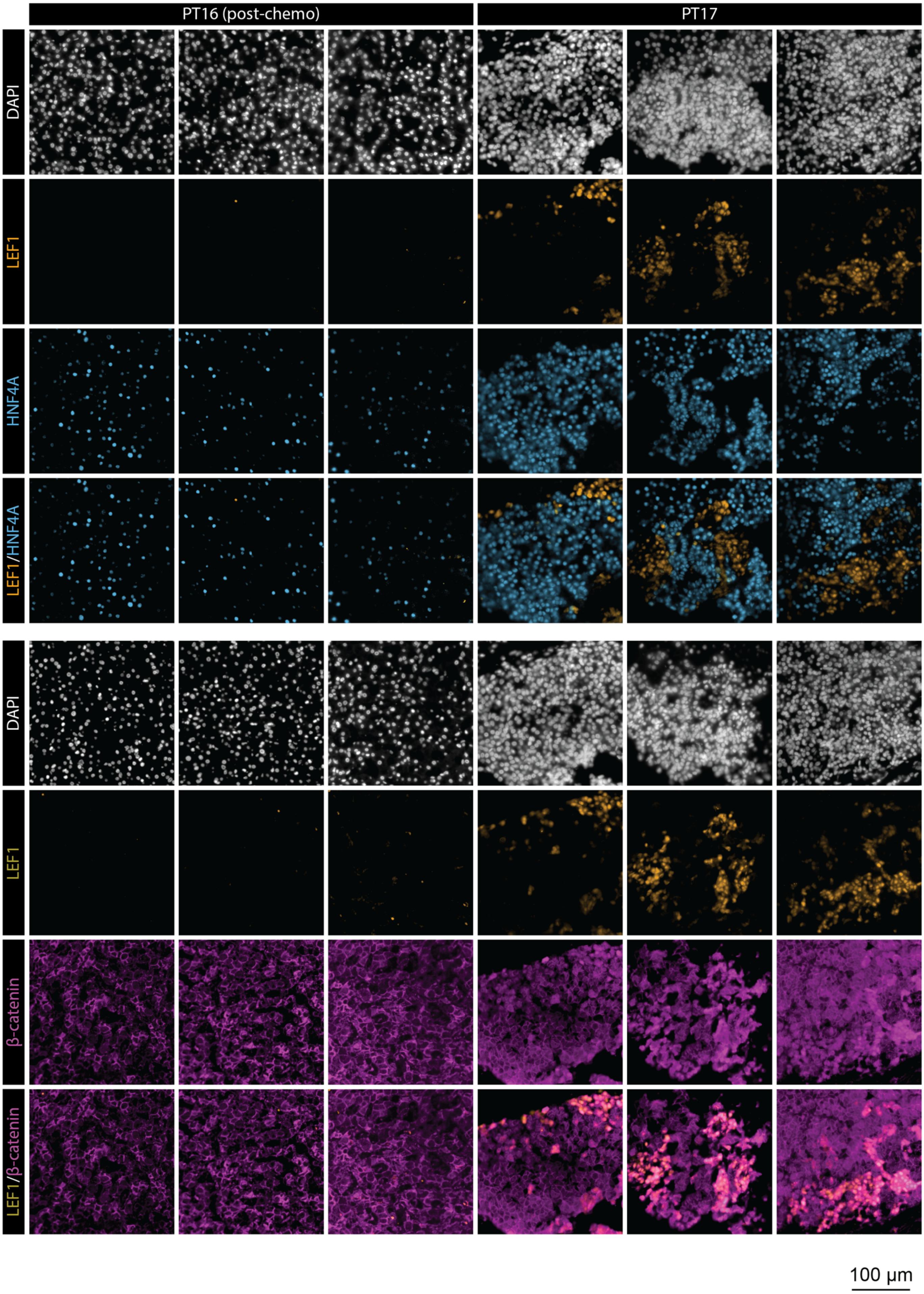
PT16 (post-chemo)
PT17
DAPI
LEF1
HNF4A
LEF1/HNF4A
DAPI
LEF1
β-catenin
LEF1/β-catenin
100 µm

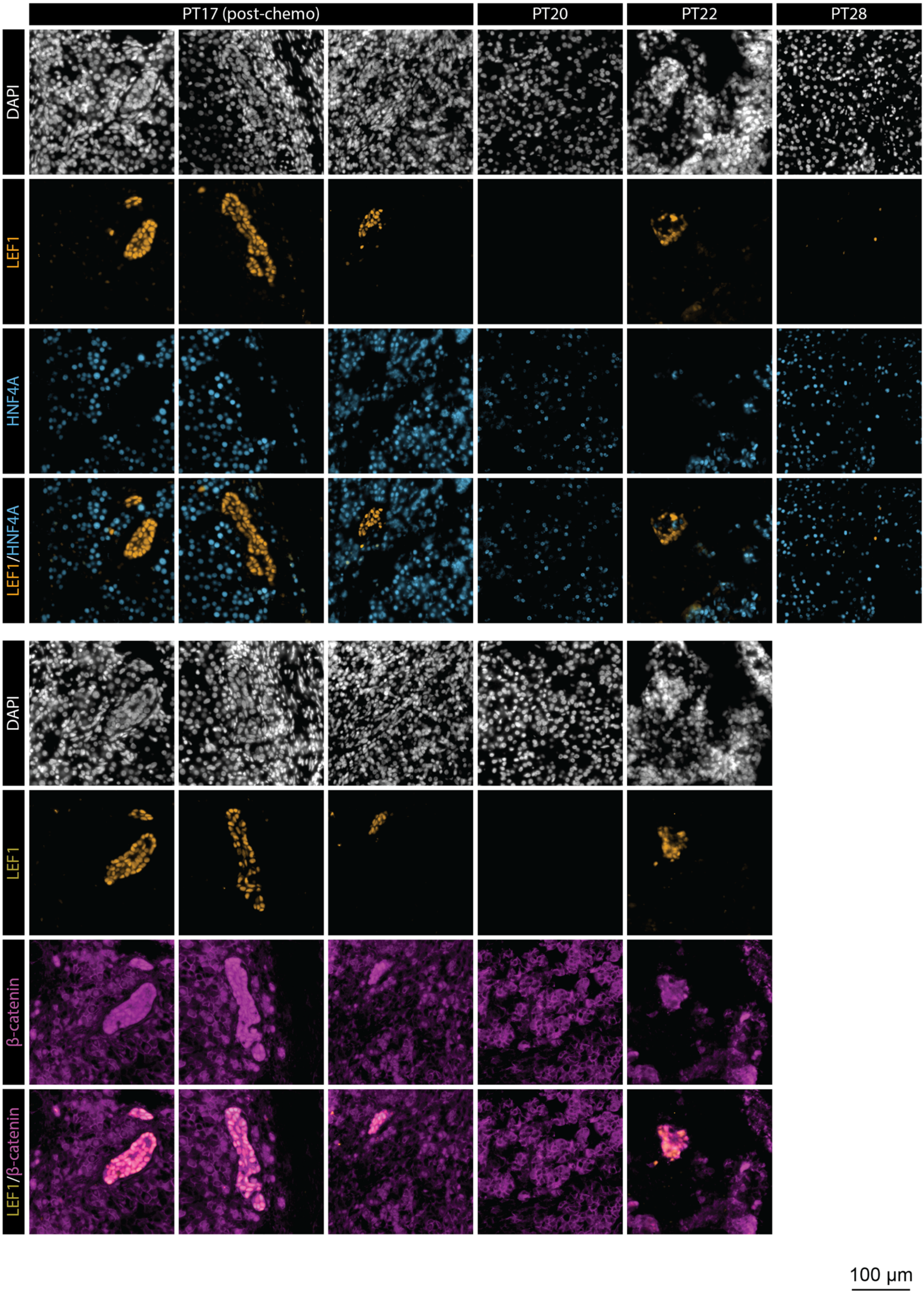
PT17 (post-chemo)
PT20
PT22
PT28
DAPI
LEF1
HNF4A
LEF1/HNF4A
DAPI
LEF1
β-catenin
LEF1/β-catenin
100 µm

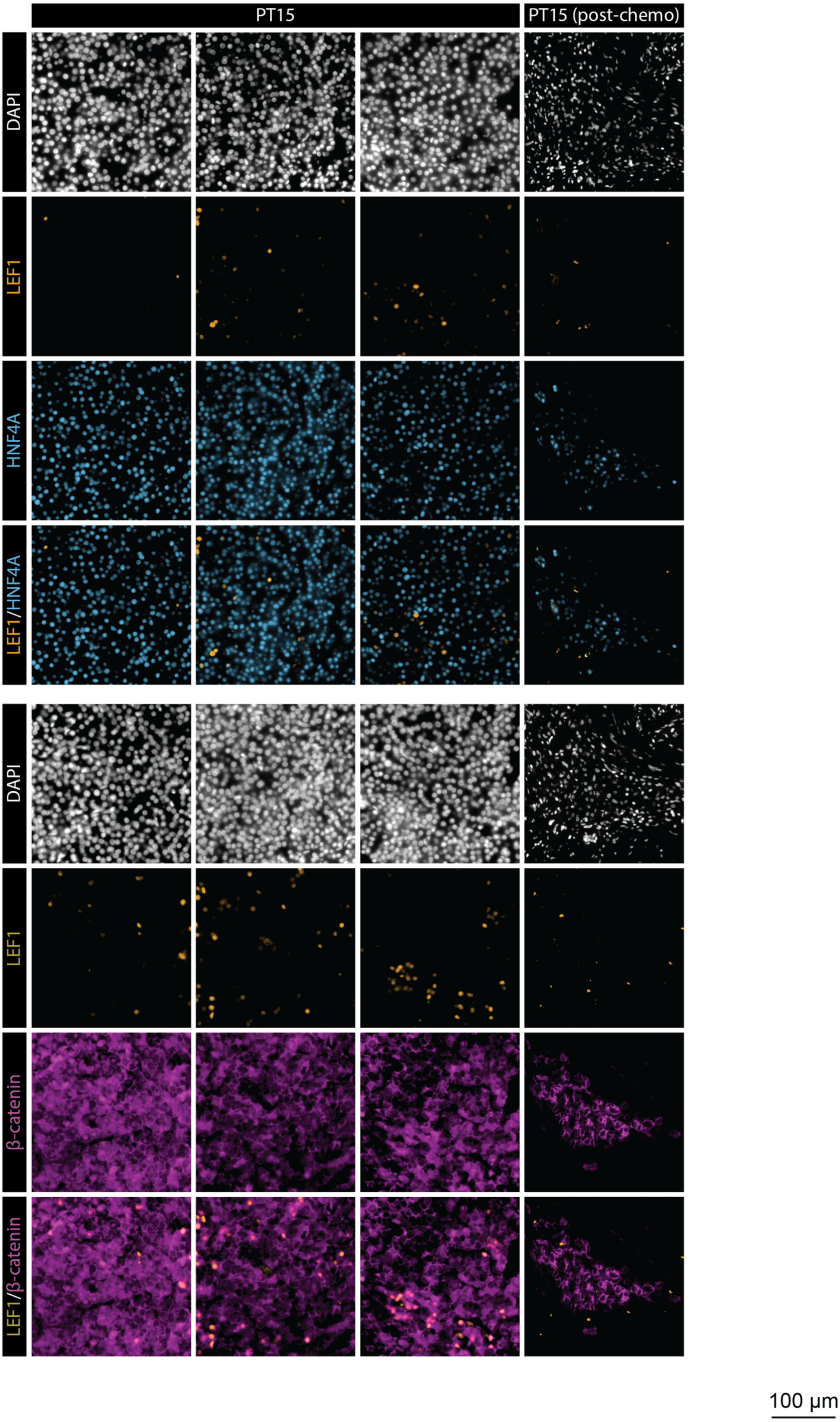

(b) Co-stainings of HNF4A/LEF1 and β-catenin/LEF1 in normal liver and tumors with fetal and embryonal regions. Co-stainings were performed on consecutive sections and the fields were selected to represent the same regions between the section. β-catenin demonstrated heterogeneity in its staining pattern compared to LEF1.

c HNF4A vs LEF1 staining intensity by sample

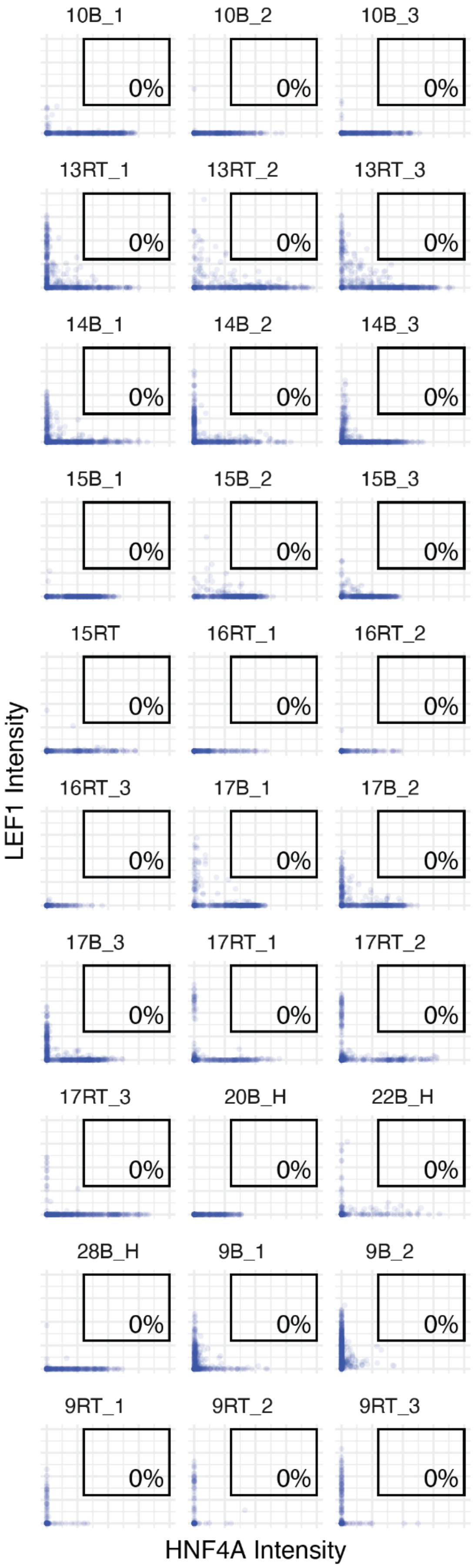

(c) Quantification of LEF1 versus HNF4A signal intensity after nuclear segmentation, per individual image of (b). Source data are provided as a Source Data file.

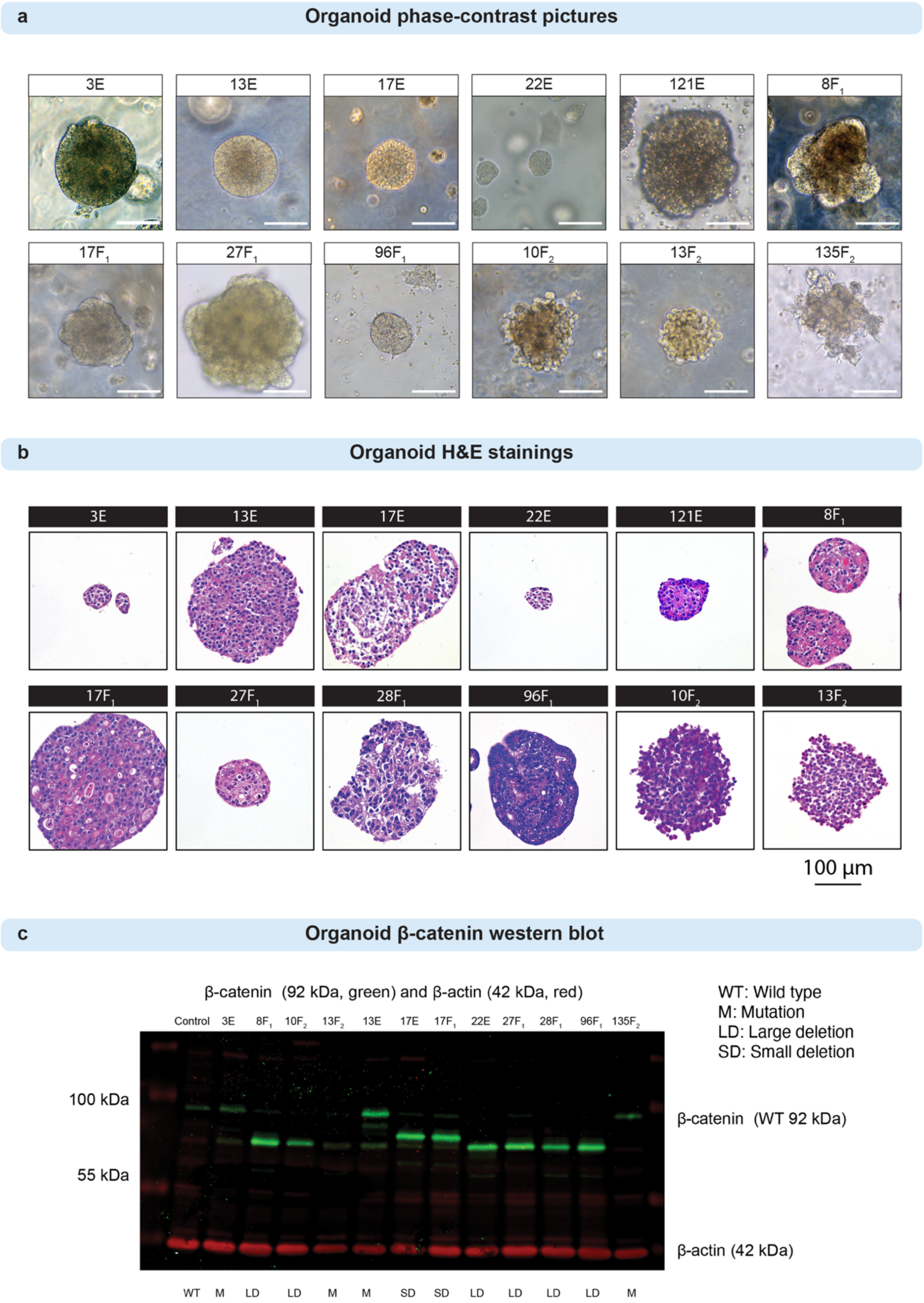

Supplementary Figure 4. Hepatoblastoma organoid analysis.

(a) Phase-contrast images of organoids. Morphologically, embryonal tumor organoids are more densely packed and have smooth surfaces while fetal tumor organoids have more irregular shapes. Scale bars, 100 µm.

(b) H&E stainings of organoids.

(c) Western blot confirms expression of mutant β-catenin proteins in organoid samples with exon 3 deletions. As positive controls, organoid samples with missense mutations and an HCC organoid sample with wild type *CTNNB1* were used. Lysates were measured in at least two independent experiments.

d **Organoid inferCNV analysis**

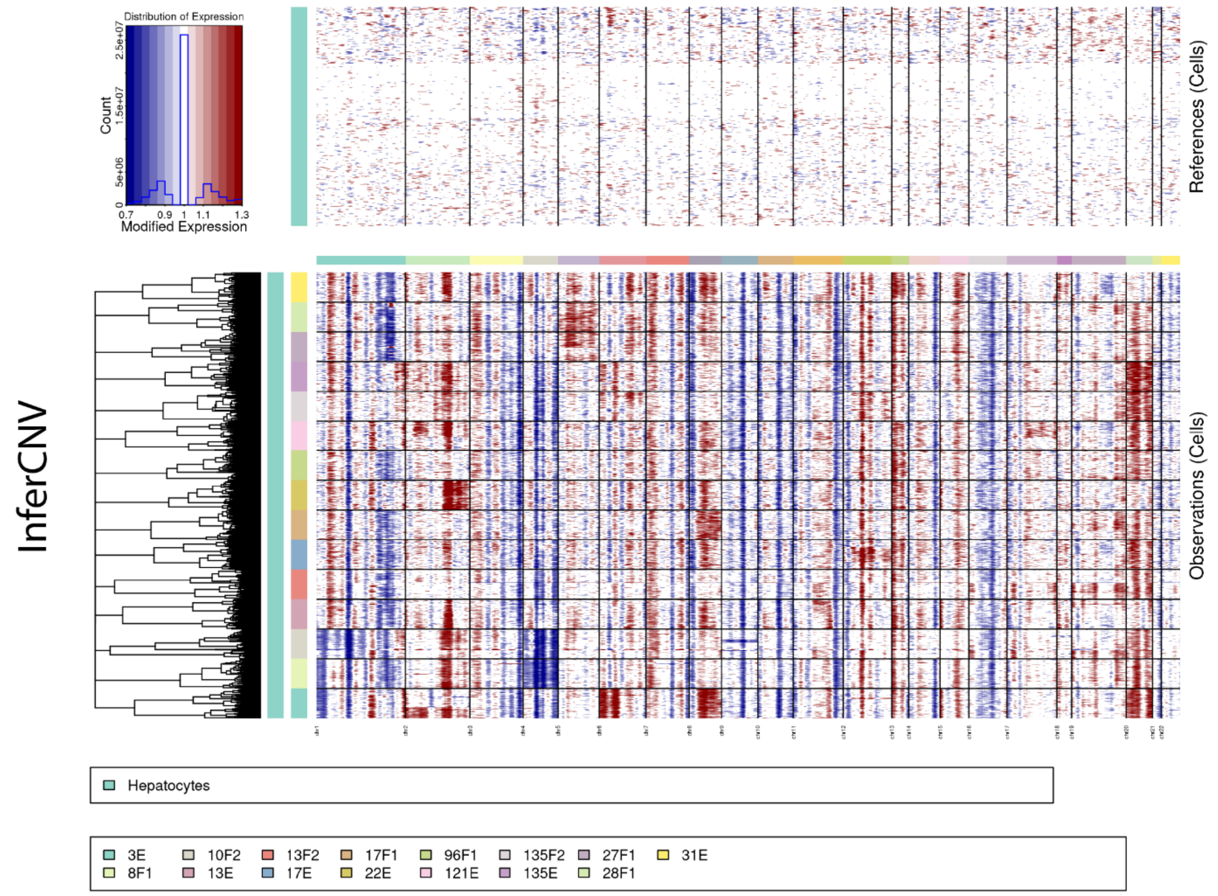

e **Organoid UMAP**

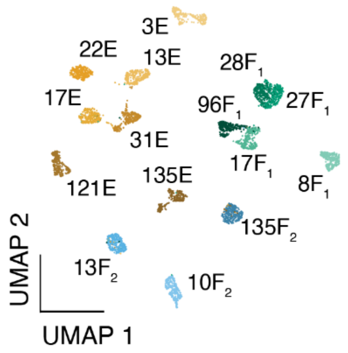

f **UMAP with low and high passage $13F_2$**

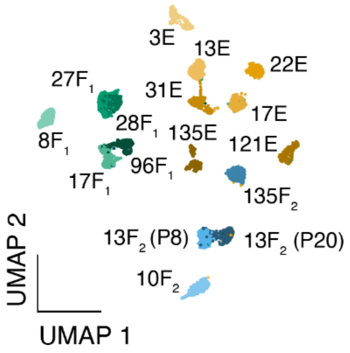

(d) InferCNV based on organoid scRNA-seq data. The hepatocyte cluster from the Song *et al.* dataset was used as reference cells.
(e) scRNA-seq UMAP of all organoids models.
(f) scRNA-seq UMAP of all organoids with low and high passage number $13F_2$.

g **Differentially expressed genes, organoid scRNA-seq**

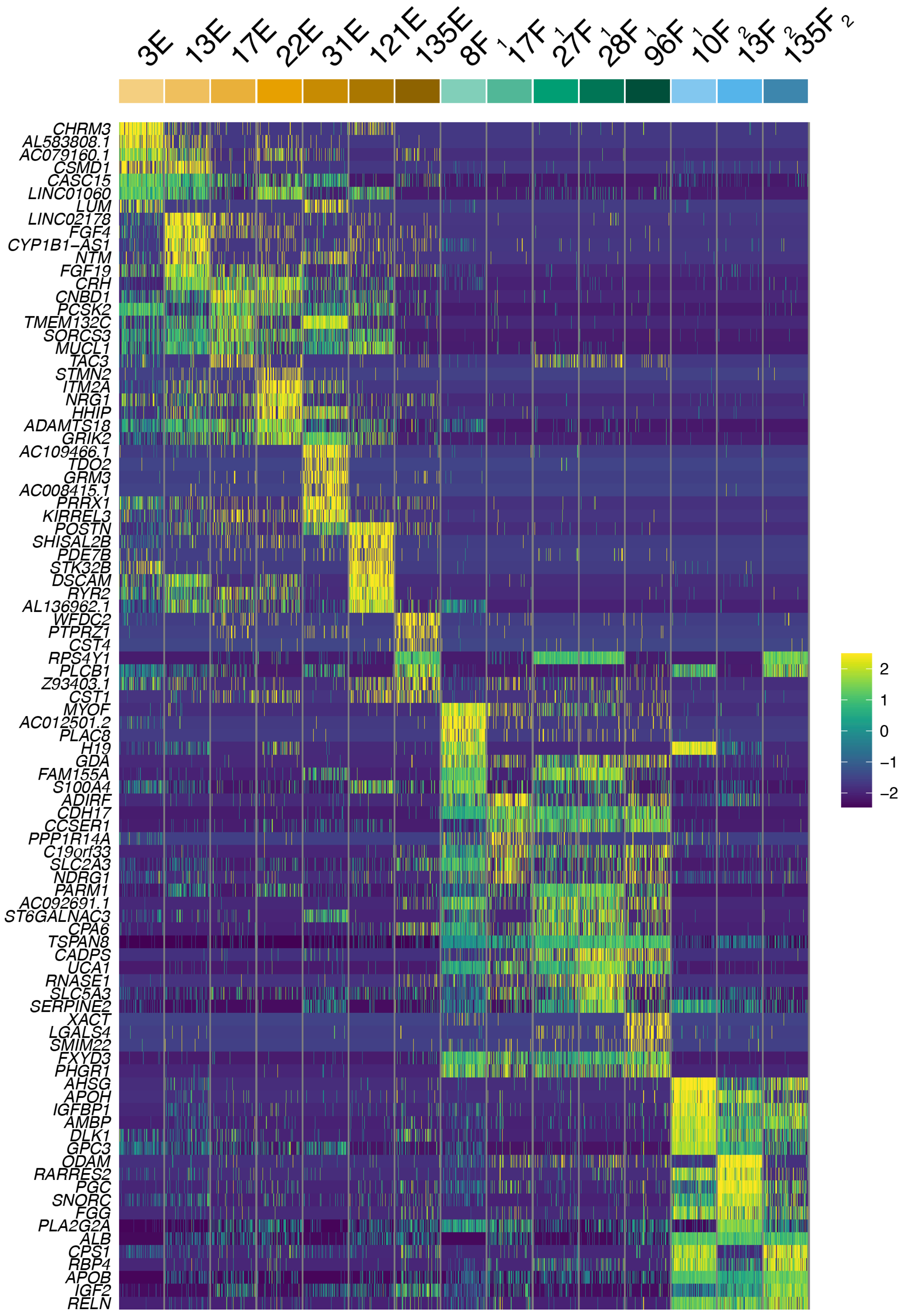

(g) Heatmap showing the top differentially expressed genes per organoid model.

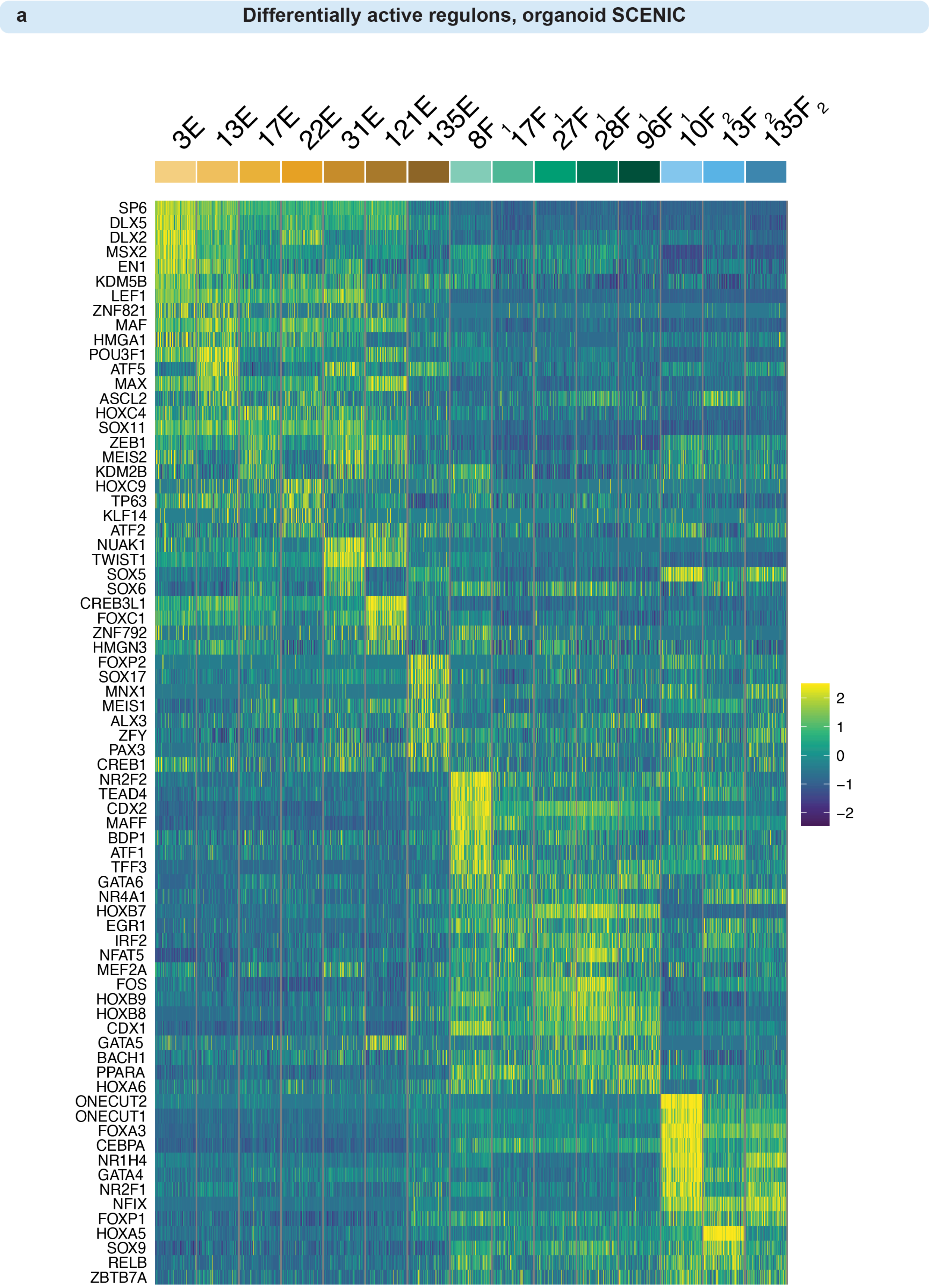

Supplementary Figure 5. Multiome and regulon analysis of hepatoblastoma organoids.
(a) Heatmap showing the top active SCENIC regulons per organoid model.

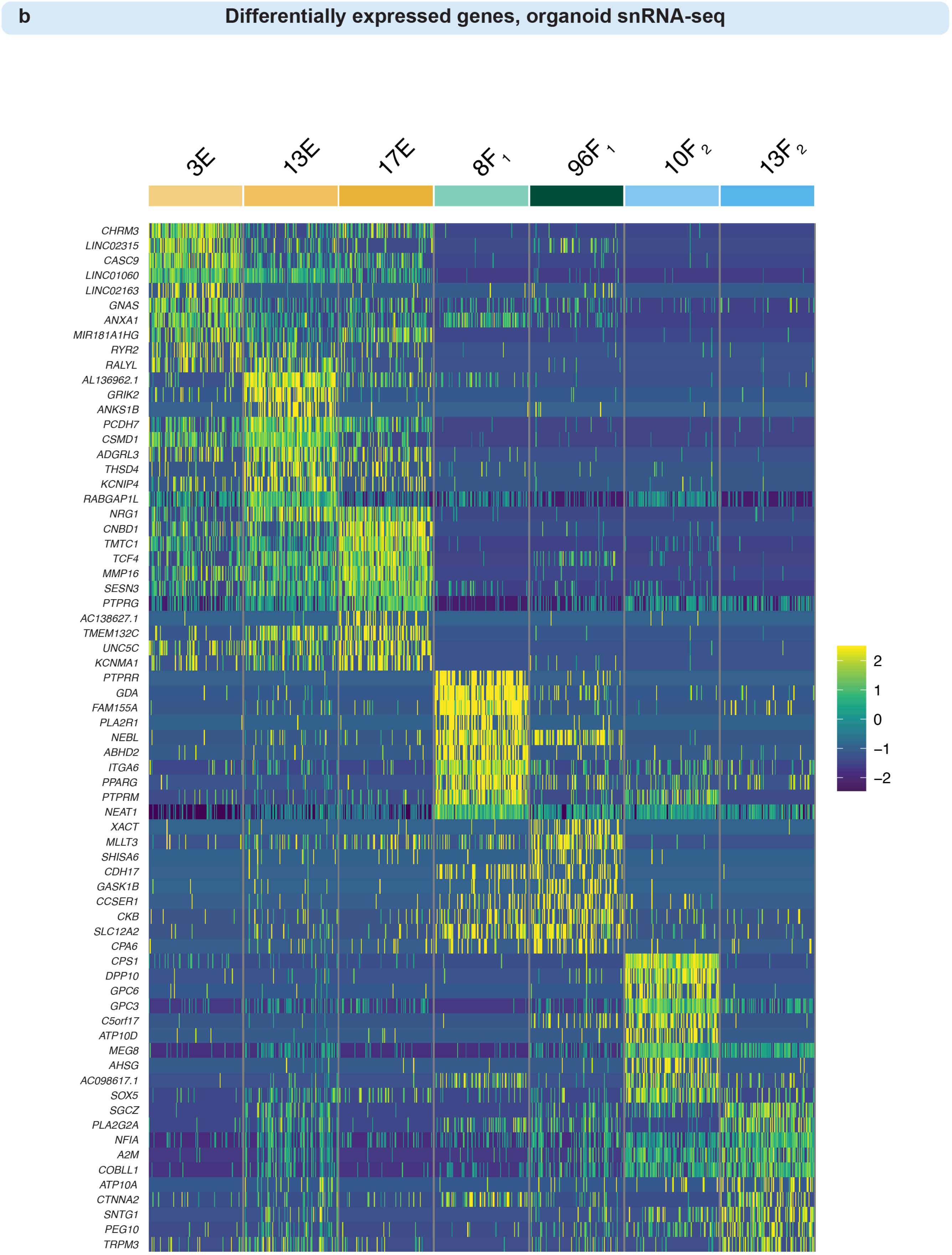

(b) Heatmap showing the top differentially expressed genes per organoid model based on the RNA counts of the single nucleus 10x Multiome dataset.

c Organoid HNF4A/LEF1 immunofluorescence staining

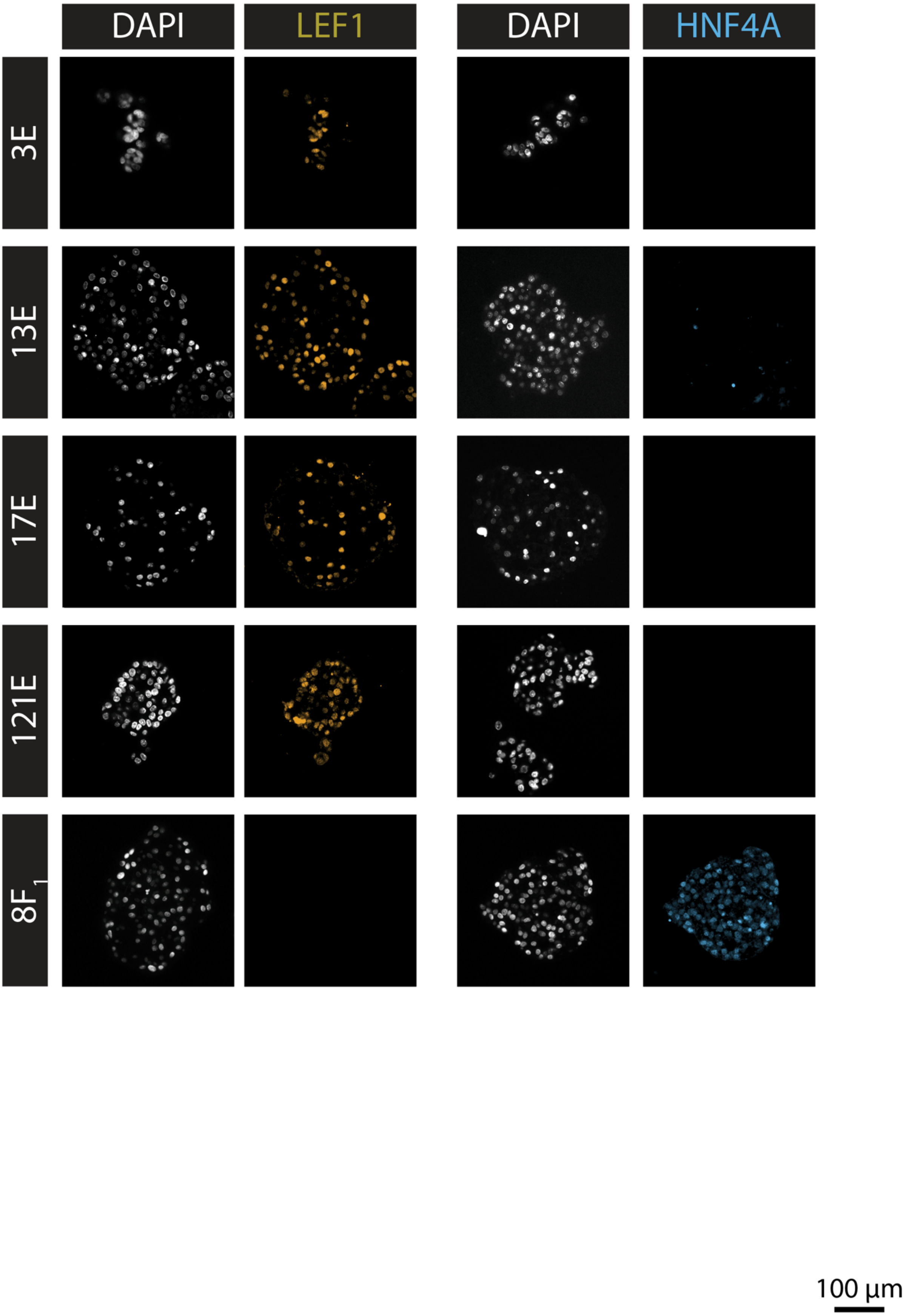

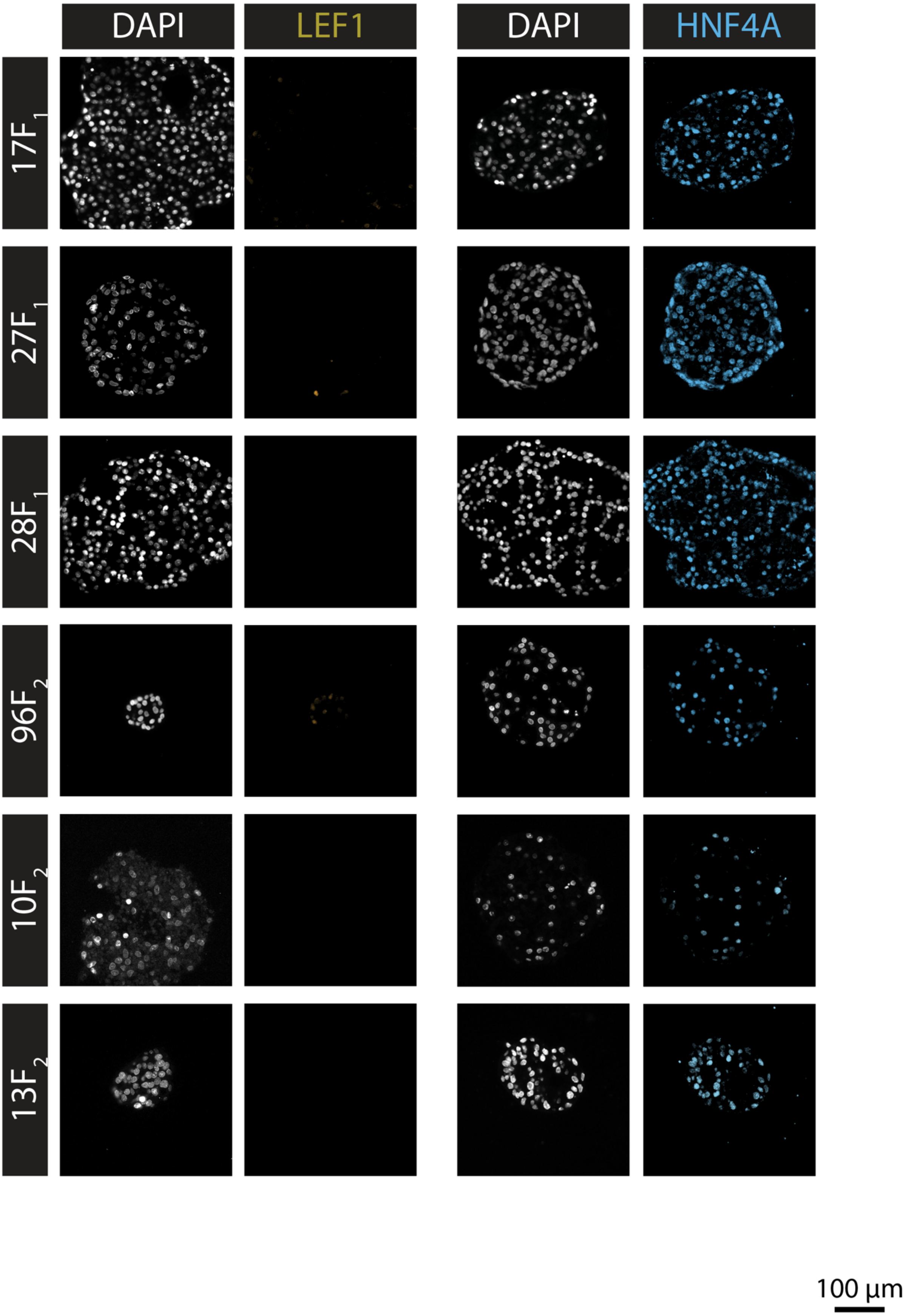

(c) IF staining of HNF4A and LEF1 in the organoid cohort, confirming the transcriptomic classification.

d Organoid β-catenin immunofluorescence staining

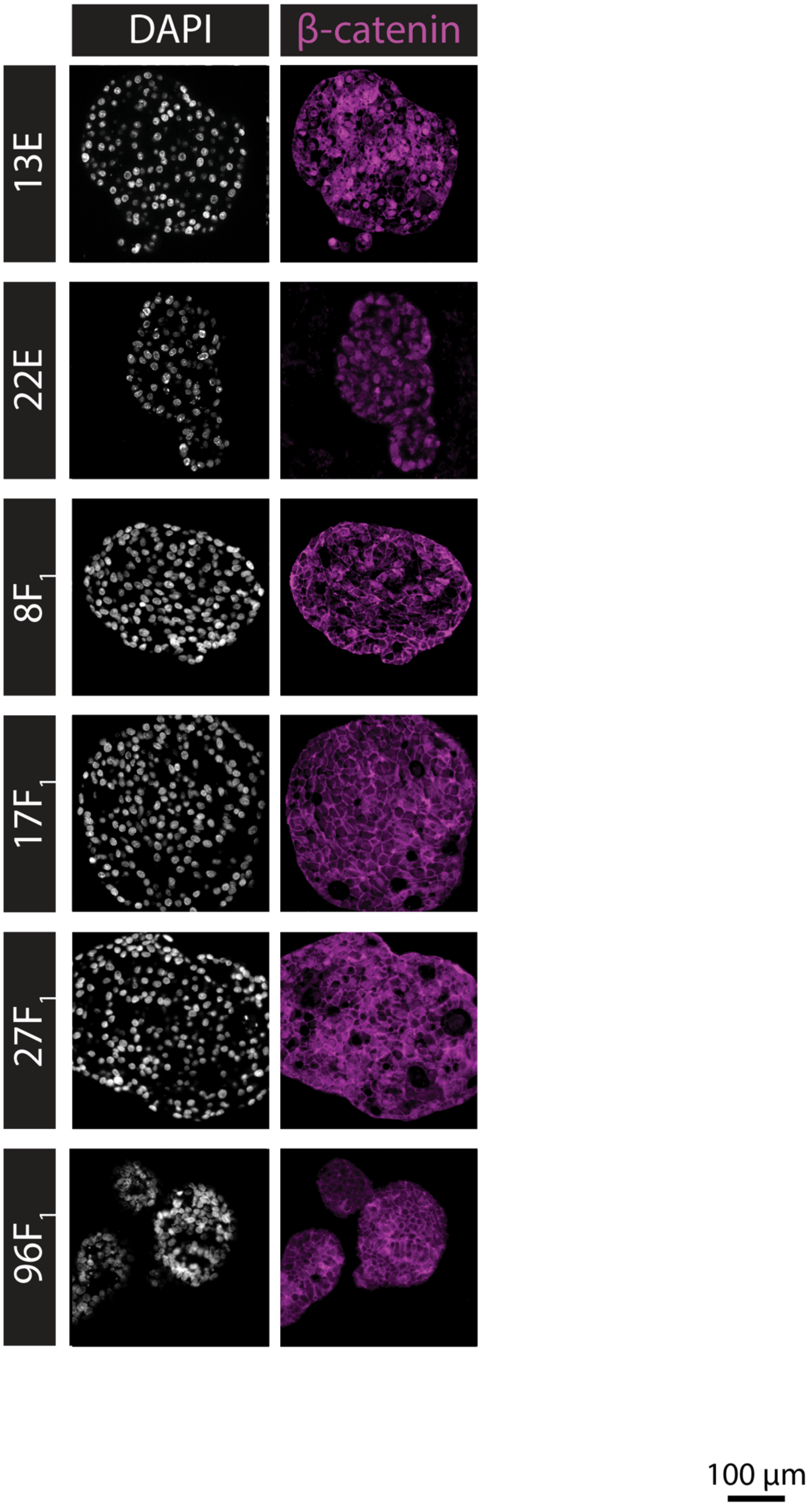

(d) IF staining of β-catenin in representative organoids.

a **Dose-response AUC heatmap**

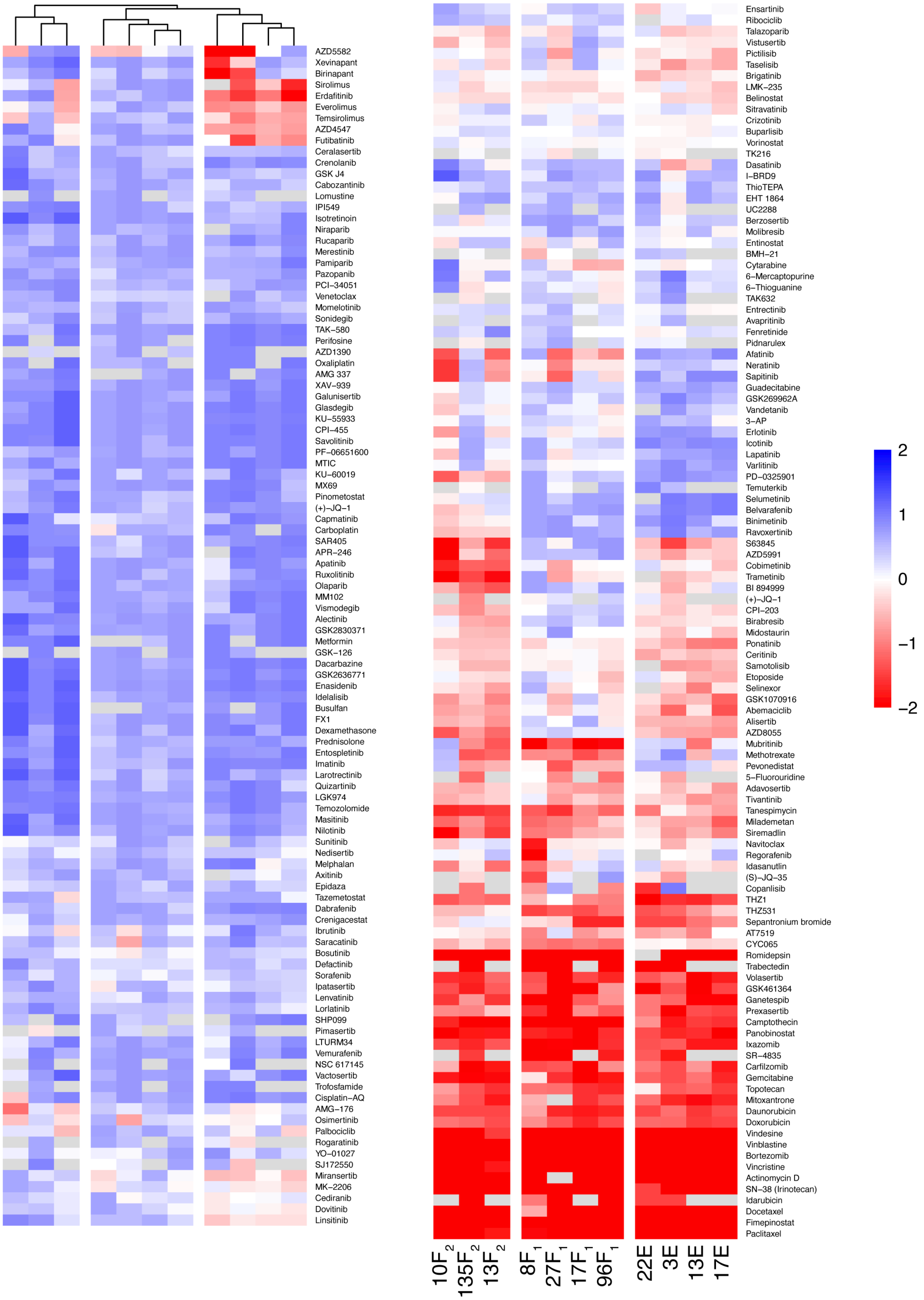

Supplementary Figure 6. High-throughput drug screening of hepatoblastoma organoids.
(a) Scaled, clustered heatmap showing AUC values for dose response curves for all compounds tested.

b Organoid drug screen dose-response curves

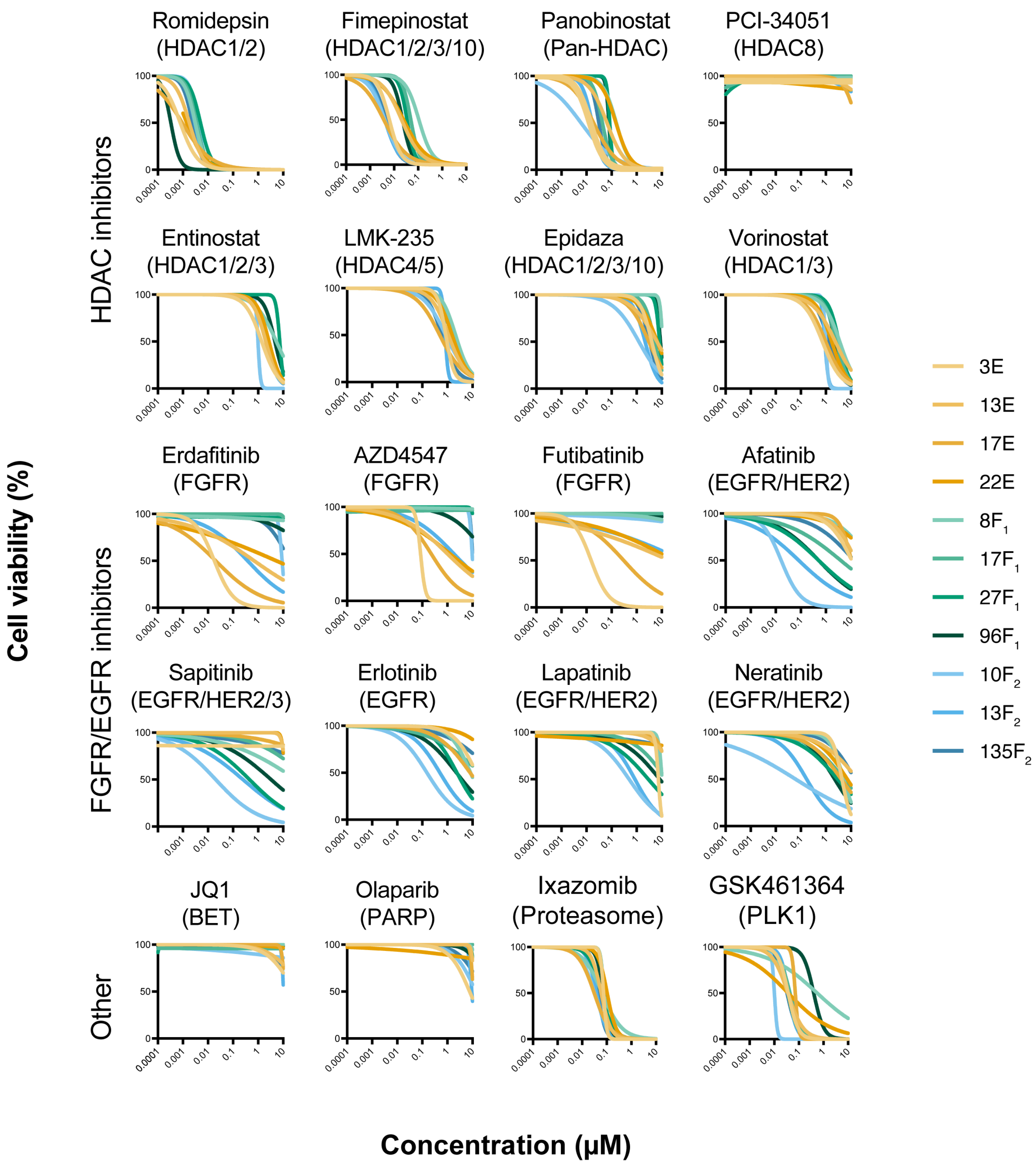

(b) Drug screening dose response curves for selected HDAC inhibitors (top), selected FGFR and EGFR inhibitors (middle) and other selected drugs (bottom).

c ***HDAC* expression**

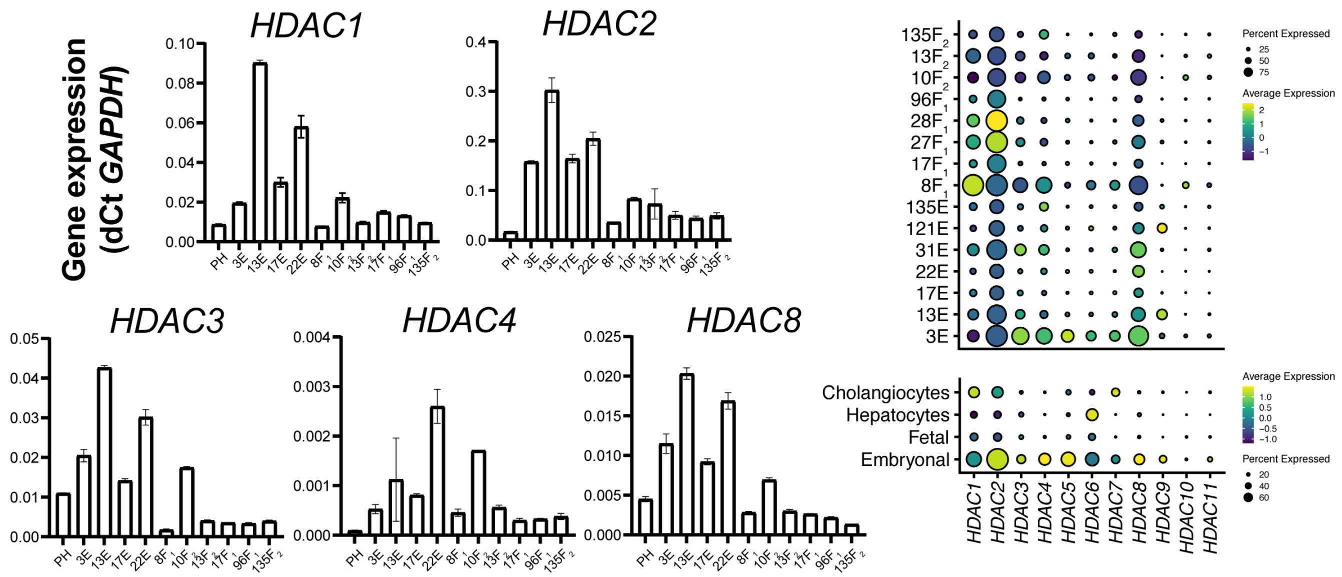

d **Organoid growth factor dependency**

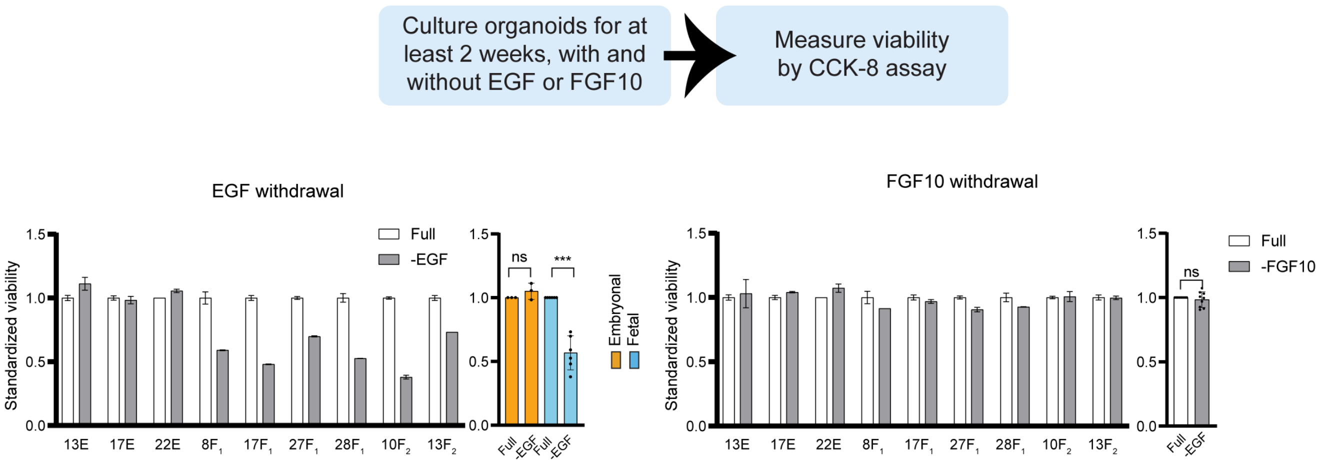

(c) qRT-PCR graphs showing expression of *HDAC* genes in different organoid models normalized against *GAPDH* (left). Error bars represent standard deviations for technical duplicates. Dot plots showing expression of all HDAC genes in organoids and tissues as measured by scRNA-seq (right).
(d) Organoid growth factor dependency per model and subgroup. Experimental setup (top) and viability assays (bottom). Error bars of single models represent standard deviations for technical duplicates. Statistical data between groups are presented as mean values with standard deviations. Statistical significance was determined using a paired two-sided t-test. ***$p < 0.001$; ns, not significant.

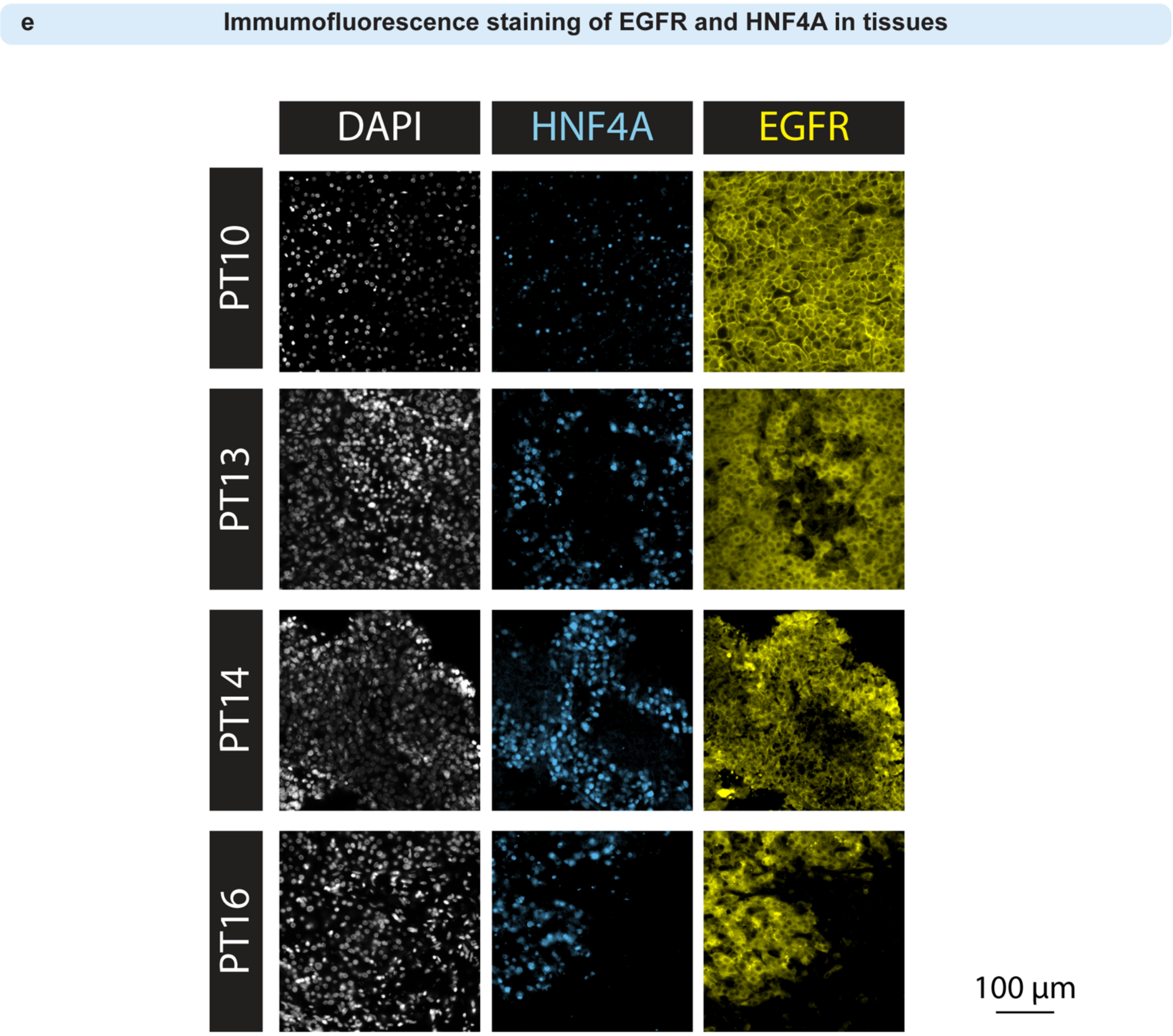

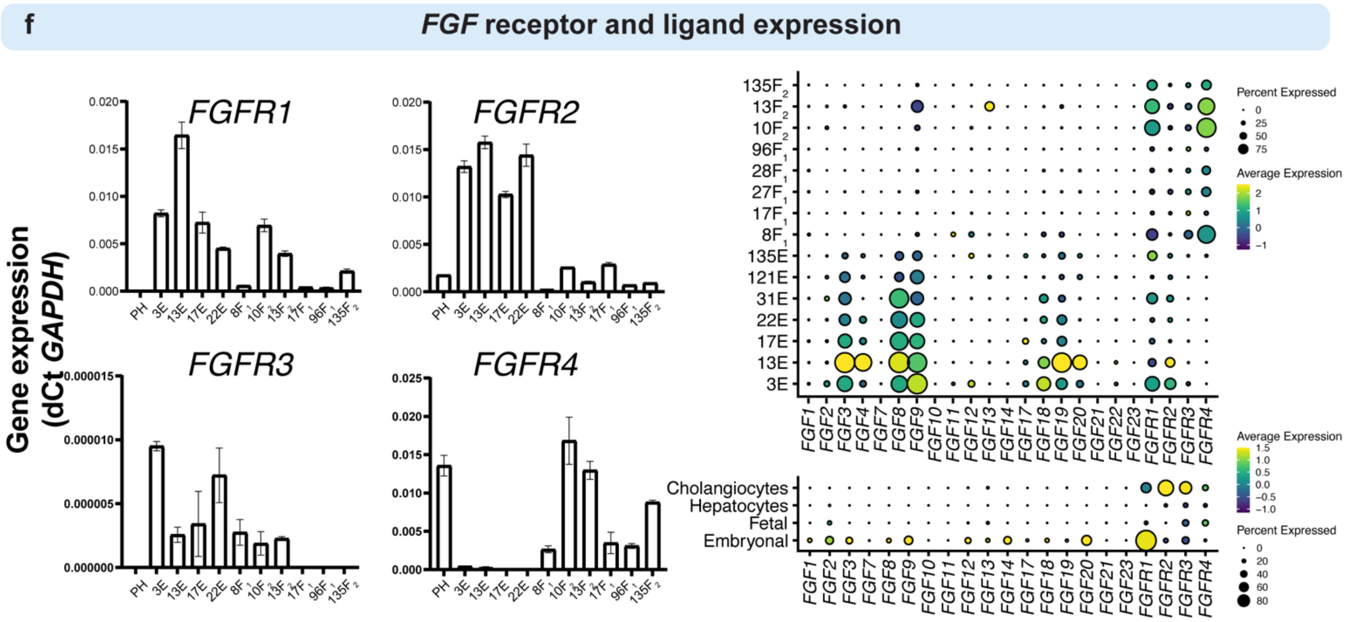

(e) Immunofluorescence co-staining of EGFR and HNF4A in selected tissues, showing EGFR expression in HNF4A+ regions.

(f) qRT-PCR graphs showing expression of *FGFR* genes in different organoid models normalized against *GAPDH* (left). Error bars represent standard deviations for technical duplicates. Dot plots showing expression of all *HDAC* genes in organoids and tissues as measured by scRNA-seq (right).

g Effect of erdafitinib (FGFRi) with and without FGF10 in the medium

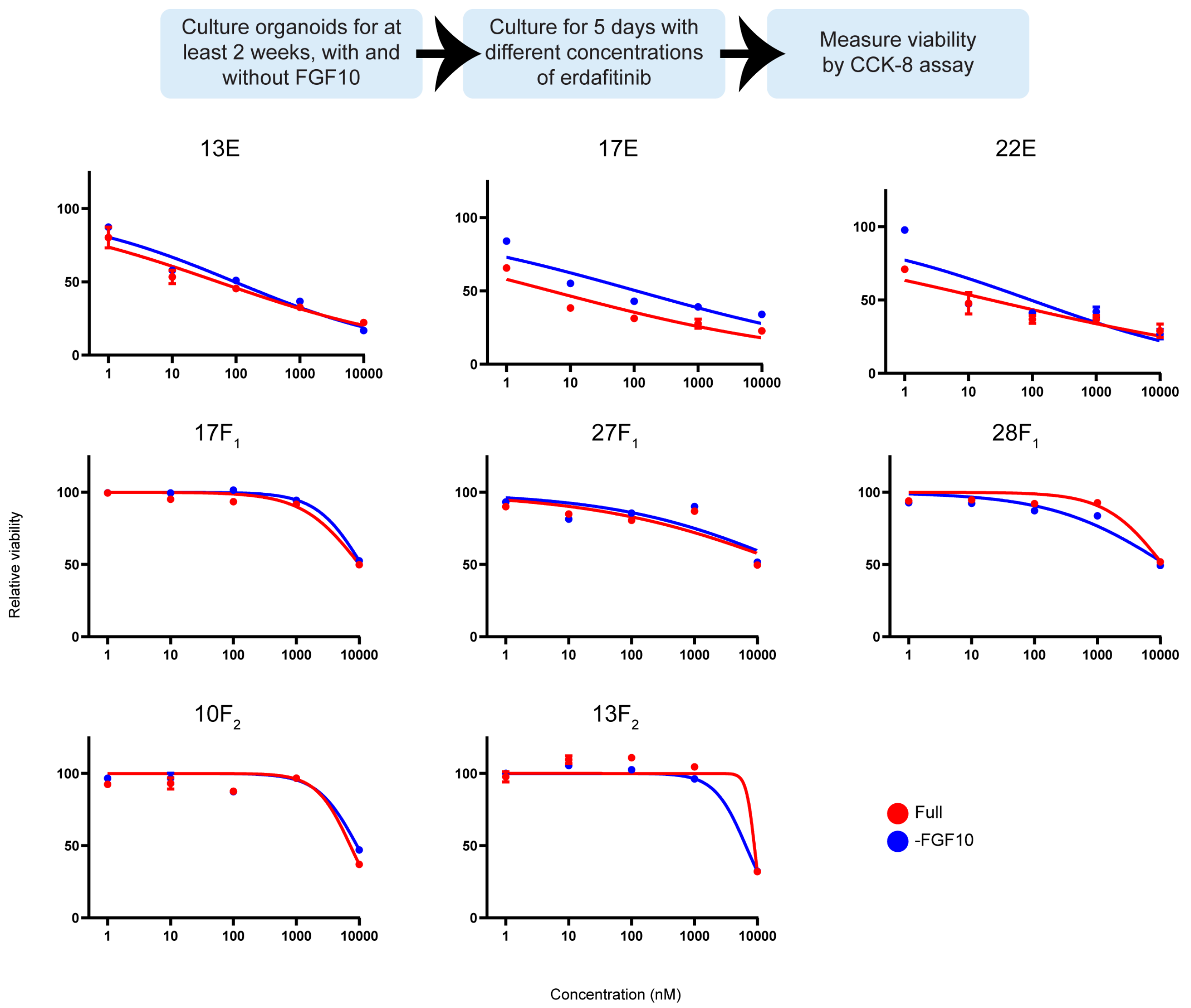

h IC50s and viability percentages at 10 μM of select drugs

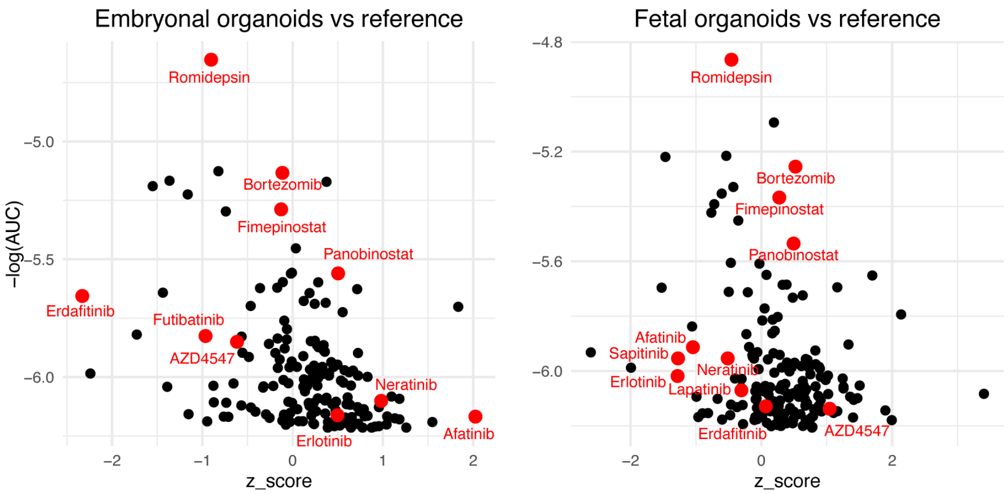

(g) Effect of erdafitinib with and without FGF10 supplemented in culture medium. Experimental setup (top). Dose response curves (bottom). Average values are plotted of technical duplicates, with error bars representing standard deviations.

(h) Hepatoblastoma organoid drug responses compared to the pediatric tumor reference cohort. Volcano plots showing the z-scores of fetal and embryonal drug responses (AUC values) versus the pediatric tumor organoid reference cohort. Lower z-scores indicate more specific sensitivity for hepatoblastoma organoids. On the y-axis, the negative logarithm of the average AUC value for the respective hepatoblastoma organoids is plotted. Only drugs with IC50 values are shown.

Source data are provided as a Source Data file.

Supplementary Table 1. Overview of hepatoblastoma patients, tissues and organoid models described in this study.

Patient characteristics					Genetics tumor biopsy		Tissue characteristics			Tissue experiments			Organoid experiments					
**Patient**	**Risk**	**Age**	**Sex**	**PRE TEXT**	***CTNNB1* (tumor AF%)**	**cnLOH 11p15**	**Material**	**Treatment**	**Histology**	**scRNA**	**Spatial**	**IF**	**ID**	**scRNA**	**snRNA**	**Screen**	**Medium**	***CTNNB1* mutation**
PT2	High	2-8y	M		Exon 3 del	UNK	Resection	Post-chemo	Predominant F		Yes							
PT3	High	<2y	F	II	p.T41A (36%)	No	Biopsy	Pre-chemo	Mixed F/E				3E	Yes	Yes	Yes	Full	Matching
PT8	Low	<2y	F	II	Exon 3 del	No	Biopsy	Pre-chemo	Predominant F				$8F_1$	Yes	Yes	Yes	Full	Matching
PT9	High	<2y	M	III	p.G34V (39%)	UNK	Biopsy	Pre-chemo	Mixed F/E/M			Yes						
							Resection	Post-chemo	Mixed F/M	Yes		Yes						
PT10	Intermediate	<2y	F	III	Exon 3 del	No	Biopsy	Pre-chemo	Predominant F			Yes	$10F_2$	Yes	Yes	Yes	Full	Matching
PT13	Very low	<2y	M	II	p.D32N (40%)	Yes	Biopsy	Pre-chemo	Mixed F/E				$13F_2$	Yes	Yes	Yes	Full	Matching
							Resection	Pre-chemo	Mixed F/E	Yes	Yes	Yes	13E	Yes	Yes	Yes	Full	UNK
PT14	Intermediate	2-8y	F	IV	p.S23_G34del (31%)	Yes	Biopsy	Pre-chemo	Mixed F/E			Yes						
							Resection	Post-chemo	Predominant F		Yes							
PT15	High	2-8y	M	IV	p.S29F; p.D32Y (37%)	No	Biopsy	Pre-chemo	Predominant F			Yes						
							Resection	Post-chemo	Predominant F			Yes						
PT16	Intermediate	<2y	M	II	Exon 3 del	UNK	Resection	Post-chemo	Mixed F/E		Yes	Yes						
PT17	High	<2y	F	II	p.V22_Q78delinsE	Yes	Biopsy	Pre-chemo	Mixed F/E			Yes	17E	Yes	Yes	Yes	Full	Matching
							Resection	Post-chemo	Mixed F/E/M			Yes	$17F_1$	Yes		Yes	Reduced	Matching
PT20	High	2-8y	M	IV	Exon 3 del	No	Biopsy	Pre-chemo	Predominant F			Yes						
PT22	Intermediate	<2y	F	I	Exon 3 del	No	Biopsy	Pre-chemo	Mixed F/E			Yes	22E	Yes		Yes	Full	Matching
PT27	High	2-8y	M	IV	Exon 3 del	UNK	Biopsy	Pre-chemo	Predominant E				$27F_1$	No		Yes	Reduced	Matching
PT28	Low	<2y	M	III	UNK	UNK	Biopsy	Pre-chemo	Mixed F/E			Yes	$28F_1$	Yes			Reduced	Matching
PT31	Intermediate	<2y	F	II	UNK	UNK	Biopsy	Pre-chemo	Mixed F/E				31E	Yes			Full	UNK
PT96	UNK	UNK	M*	UNK	UNK	UNK	Resection	Post-chemo	UNK				$96F_1$	Yes	Yes	Yes	Reduced	Exon 3 del
PT121	UNK	UNK	F*	UNK	UNK	UNK	Relapse (lung metastasis)	Post-chemo	UNK				121E	Yes			Full	p.D32N
PT135	UNK	UNK	M*	UNK	UNK	UNK	Relapse	Post-chemo	UNK				$135E/F_2$	Yes		Yes	Full	p.S29F; p.D32Y

Notes: cnLOH 11p15 = copy neutral loss of heterozygosity of chr11p15.5; UNK = Unknown; F = fetal, E = embryonal, M = mesenchymal; asterisks (*) indicates sex imputed based on organoid scRNA-seq analysis. **Tumor origin of the organoids was confirmed based on *CTNNB1* mutation (Sanger sequencing) or expression of truncated β-catenin protein (western blotting). Viably frozen tumor samples PT96, PT121 and PT135 were obtained from an external source and no matching FFPE tissues were available for these samples.

Supplementary Table 2. IC50s and viability percentages at 10 μM of select drugs.

	**IC50 E (μM)**	**IC50 F (μM)**	**Endpoint E (%)**	**Endpoint F (%)**
Romidepsin	0.001 ± 0.000	0.003 ± 0.001	2 ± 3	2 ± 2
Fimepinostat	0.013 ± 0.010	0.032 ± 0.037	4 ± 2	4 ± 1
Panobinostat	0.062 ± 0.063	0.059 ± 0.051	6 ± 2	5 ± 2
Volasertib	0.053 ± 0.014	0.084 ± 0.078	3 ± 2	13 ± 17
Bortezomib	0.007 ± 0.001	0.018 ± 0.025	5 ± 2	4 ± 2
AZD4547	0.653 ± 0.588	>10	17 ± 10	68 ± 29
Erdafatinib	1.520 ± 2.571	>10	21 ± 22	67 ± 34
Afatinib	>10	>10	60 ± 11	33 ± 25
Sapitinib	>10	>10	81 ± 10	47 ± 25
Erlotinib	>10	>10	66 ± 16	36 ± 23
Lapatinib	>10	>10	63 ± 33	42 ± 29
Neratinib	8.435 ± 6.676	4.247 ± 4.794	38 ± 19	29 ± 21

Notes: Average IC50 values and endpoint viability percentages for all tested FGFR and EGFR inhibitors, ± standard deviation.

Supplementary Table 3. Reference cohort drug screening.

Sample ID	Diagnosis group	Research group
01-017	Neuroblastoma	Molenaar
RMS109	Rhabdomyosarcoma	Drost
JD62T	Wilms Tumor	Drost
RMS110	Rhabdomyosarcoma	Drost
RMS000HQC	Rhabdomyosarcoma	Drost
RMS000FLV	Rhabdomyosarcoma	Drost
MRT_JD81T	Malignant Rhabdoid Tumor	Drost
RMS000HWQ	Rhabdomyosarcoma	Drost
AMC753	Neuroblastoma	Molenaar
RMS007	Rhabdomyosarcoma	Drost
RMS000EEC	Rhabdomyosarcoma	Drost
RWT_119T	Wilms Tumor	Drost
RWT_77T	Wilms Tumor	Drost
01-182	Neuroblastoma	Molenaar
ES-046	Ewing Sarcoma	Clevers
AMC717	Neuroblastoma	Molenaar
ES-041	Ewing Sarcoma	Clevers
OVT-054	DSCRT	Clevers
2.0_066	Neuroblastoma	Molenaar
2.0_072	Neuroblastoma	Molenaar
RWT_117T	Wilms Tumor	Drost
2.0_093	Neuroblastoma	Molenaar
JD104T	Wilms Tumor	Drost
RWT_125T	Wilms Tumor	Drost
RWT_126T	Wilms Tumor	Drost
RWT_136T	Wilms Tumor	Drost
RWT_123T	Wilms Tumor	Drost

Supplementary Table 4. Organoid passage numbers

Line	scRNA-seq	snRNA-seq	IF staining	Drug screen
3E	5	9	10	6
8F1	4	10	4	4
10F2	7	7	6	9
13F2	8 & 20	8	6	11
13E	3	11	8	7
17E	3	10	5	3
17F1	2	NA	3	6
22E	5	NA	4	4
27F1	4	NA	4	3
28F1	4	NA	3	NA
31E	3	NA	NA	NA
96F1	4	0	2	3
121E	3	NA	2	NA
135	4	NA	NA	4

Supplementary Table 5. Primary antibodies.

Target	Supplier	Dilution
HNF4A	MA1-199 (Thermo Fisher)	1:50
	sc-8987 (Santa Cruz)	1:50
b-catenin	610154 (BD Bioscience)	1:100
	8480 (Cell Signaling Tech)	1:100
LEF1	2230 (Cell Signaling Tech)	1:100
EGFR	4267 (Cell Signaling Tech)	1:100

Supplementary Table 6. Primer sequencing.

Assay	Primer	Primer sequence
Sanger sequencing	CTNNB1_Ex3_Forward	AGCGTGGACAATGGCTACTCAA
Sanger sequencing	CTNNB1_Ex3_Reverse	ACCTGGTCCTCGTCATTTAGCAGT
qRT-PCR	GAPDH Forward	CCACCTTTGACGCTGGG
qRT-PCR	GAPDH Reverse	CATACCAGGAAATGAGCTTGACA
qRT-PCR	HDAC1 Forward	CTACTACGACGGGGATGTTG
qRT-PCR	HDAC1 Reverse	GAGTCATGCGGATTCGGTGAG
qRT-PCR	HDAC2 Forward	AATCCGTAATGTTGCTCGA
qRT-PCR	HDAC2 Reverse	CATTATATGGCAACTCATTGGG
qRT-PCR	HDAC3 Forward	CCTGGCATTGACCCATAGCC
qRT-PCR	HDAC3 Reverse	CTCTTGGTGAAGCCTTGCATA
qRT-PCR	HDAC4 Forward	CGGTCCTGGGAATGTACGAC
qRT-PCR	HDAC4 Reverse	GGCCACTTTCTGCTTTAGCCT
qRT-PCR	HDAC8 Forward	TCGCTGGTCCCGGTTTATATC
qRT-PCR	HDAC8 Reverse	TACTGGCCCGTTTGGGGAT
qRT-PCR	FGFR1 Forward	GAGCCTTGTCACCAACCTCTAAC
qRT-PCR	FGFR1 Reverse	CCCAGGGCTGGGCTTGTT
qRT-PCR	FGFR2 Forward	GCCGTGAAGATGTTGAAAGATGA
qRT-PCR	FGFR2 Reverse	GTGTGCAGGCTCCAAGAAGA
qRT-PCR	FGFR3 Forward	GGTCGCACGGACGCA
qRT-PCR	FGFR3 Reverse	GCTCGGGAGACTGGCG
qRT-PCR	FGFR4 Forward	TATCTGGAGTCCCGGAAGTGTATC
qRT-PCR	FGFR4 Reverse	CAGCCCAAAGTCAGCAATCTTC
