# Supplementary material for: Divergent WNT signaling and drug sensitivity profiles within hepatoblastoma tumors and organoids
Source: Nat Commun. 2024 Nov 20;15:8576. doi: 10.1038/s41467-024-52757-w (PMC11579375; doi:10.1038/s41467-024-52757-w)
Supplement: Supplementary file 2 — Description of Additional Supplementary Files [file 41467_2024_52757_MOESM2_ESM.pdf]

Supplementary Data 1. Hepatoblastoma transcriptomic signature gene lists from our re-analysis of the Song *et al.*[21] dataset showing the top significantly differentially expressed genes between our fetal and embryonal tumor populations (adjusted *p*-value < 0.05, greatest log2FC values) and the different hepatoblastoma transcriptomic classifications were compiled from existing literature[14,17,18,21,30].

Supplementary Data 2. All compounds comprising the drug screening library shown in Fig. 6 and Supp. Fig. 6.

Supplementary Data 3. General WNT genes curated from all WNT signaling pathway collections in GOBP, KEGG, PID and Hallmarks (MSigDB v2023.2Hs), in addition to manually accrued WNT genes.
